# Antibiofilm and Anti-Hyphal Activities of Halogenated Benzophenones Against Azole-Resistant *Candida albicans*

**DOI:** 10.3390/ijms27177528

**Published:** 2026-08-22

**Authors:** Juyeon Jo, Ziyad Abdelaal, Yong-Guy Kim, Jin-Hyung Lee, Jintae Lee

**Affiliations:** 1 School of Chemical Engineering, Yeungnam University, 280 Daehak-Ro, Gyeongsan 38541, Republic of Korea; dusdl6631@yu.ac.kr (J.J.); zabdelaal@yu.ac.kr (Z.A.); 2 Institute of Clean Technology, Yeungnam University, 280 Daehak-Ro, Gyeongsan 38541, Republic of Korea; yongguy7@ynu.ac.kr

**Keywords:** anti-virulence strategy, benzophenone derivatives, biofilm formation, *Candida albicans*, hyphal transition

## Abstract

*Candida albicans* biofilms are a major cause of persistent infections and contribute to antifungal resistance as well as limitations in drug delivery. Targeting virulence traits such as biofilm formation and hyphal transition represents an effective strategy for controlling fungal pathogenicity without exerting strong selective pressure on planktonic growth. In this study, a library of structurally diverse benzophenone derivatives was screened to identify compounds with antibiofilm and anti-hyphal activities against azole-resistant *C. albicans*. Most benzophenone derivatives exhibited weak antifungal activity (MIC ≥ 200 µg/mL). However, several halogenated benzophenones markedly suppressed biofilm formation. Among them, decafluorobenzophenone at 10 µg/mL displayed the strongest inhibition, reducing biofilm formation to approximately 1–2% of control levels while maintaining substantial planktonic cell viability. Microscopy confirmed hyphal suppression, while qRT-PCR showed a 36-fold reduction in *ALS3* expression. These findings indicate that multi-halogenated benzophenones act primarily as anti-virulence agents targeting biofilm formation and hyphal development. Molecular docking suggested a possible interaction of decafluorobenzophenone with the Als3 binding pocket. Decafluorobenzophenone showed low toxicity, with unaffected *Caenorhabditis elegans* viability at 10 µg/mL, plant germination at 100 µg/mL, and only slight hemolysis at 100 µg/mL. The results highlight halogen substitution as a key structural determinant and identify benzophenone scaffolds as promising leads for developing novel antibiofilm strategies against azole-resistant *Candida* infections.

## 1. Introduction

*Candida albicans* is an important opportunistic fungal pathogen responsible for both superficial and systemic infections, particularly in immunocompromised patients. The treatment of candidiasis has become increasingly challenging due to the limited number of antifungal agents and the rapid emergence of resistance, especially to azole-based drugs [1]. One of the major causes of therapeutic failure is the ability of *C. albicans* to form biofilms on host tissues and medical devices. Biofilm-associated cells exhibit significantly higher tolerance to antifungal agents than planktonic cells, leading to persistent and recurrent infections [2]. Therefore, suppression of biofilm formation has emerged as a promising strategy for improving antifungal efficacy [3]. Among non-albicans *Candida* species, *Candida parapsilosis* is also an important opportunistic pathogen, and biofilm-forming *C. parapsilosis* isolates have been associated with significantly higher mortality rates [4].

Biofilm development in *C. albicans* is a complex and tightly regulated process involving adhesion, yeast-to-hypha transition, and maturation into structured communities. Among these steps, hyphal morphogenesis plays a critical role in adhesion, invasion, and biofilm architecture. Inhibition of hyphal formation has been shown to significantly impair biofilm development and reduce pathogenicity [5,6]. Accordingly, targeting virulence-associated processes such as filamentation and adhesion, rather than directly inhibiting cell growth, has gained attention as a complementary strategy to combat fungal infections and reduce resistance pressure [7,8].

Benzophenone and its derivatives represent a versatile class of bioactive molecules with diverse pharmacological properties, including antibacterial, antifungal, anti-inflammatory, and anticancer activities. Several synthetic benzophenone analogues have shown measurable antifungal activity against *Candida* and other fungal species, and structural modifications such as incorporation of morpholine or triazole moieties have been reported to enhance antifungal potency [9,10,11]. In addition, naturally occurring benzophenone-related compounds have been shown to inhibit germ tube formation, suppress hyphal development, and interfere with biofilm maturation, suggesting that this scaffold can modulate virulence-associated pathways in *C. albicans* [12,13].

Previous structure–activity relationship studies have shown that halogen substitution can enhance the biological and antimicrobial activities of benzophenone derivatives [10,14,15]. In particular, recent reviews have emphasized that halogenation is an important strategy in medicinal chemistry because it can improve membrane permeability, hydrophobic interactions, molecular stability, and target-binding affinity, thereby enhancing the biological and antimicrobial activities of halogenated compounds [16]. Recent studies have further demonstrated that halogenated compounds can suppress adhesion, filamentation, and biofilm-associated pathways in *C. albicans*, suggesting an important role of halogen substitution in regulating fungal virulence [17]. Despite these advances, the antibiofilm and anti-hyphal activities of structurally diverse and multi-halogenated benzophenone derivatives against azole-resistant *C. albicans* have not been systematically investigated.

Therefore, in the present study, a structurally diverse benzophenone library was screened to identify inhibitors of biofilm formation and hyphal transition. To better interpret the screening results, the 101 commercially available benzophenone derivatives were structurally classified into four groups: non-halogenated, mono-halogenated, multi-halogenated, and fluorine-rich derivatives. This classification allowed an exploratory evaluation of how halogenation status and substitution patterns are associated with antibiofilm and anti-hyphal activities against *C. albicans*. The antibiofilm activity was also evaluated against *C. parapsilosis* as an additional non-albicans *Candida* species. In addition to antibiofilm screening, microscopic analysis, molecular docking, and toxicity evaluation were performed to investigate the anti-virulence potential and structure–activity relationships of halogenated benzophenone derivatives.

## 2. Results

### 2.1. Benzophenone Derivative Library Screening Identifies Decafluorobenzophenone as a Potent Antibiofilm Lead Against Azole-Resistant C. albicans

**Figure 1:**
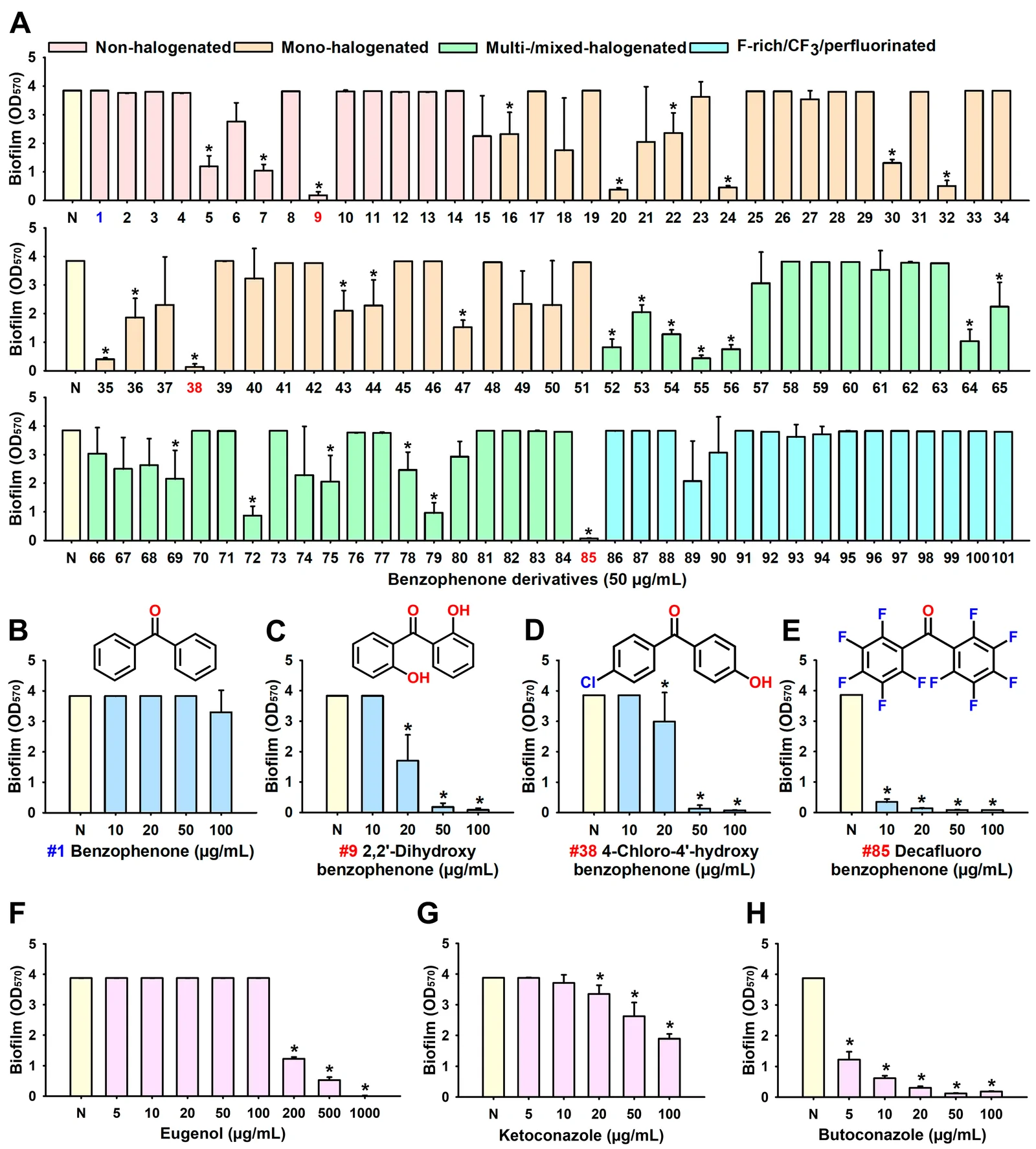
Antibiofilm activity of halogenated benzophenone derivatives against *Candida albicans***.** The antibiofilm efficacy of 101 benzophenone derivatives against azole-resistant *C. albicans* DAY185 was evaluated at a final concentration of 50 µg/mL (**A**). In panel A, pink, orange, green, and blue indicate 15 non-halogenated derivatives, 36 mono-halogenated derivatives, 33 multi-halogenated derivatives, and 17 fluorine-rich derivatives, respectively. Dose-dependent inhibition of biofilm formation by selected compounds, #1 (backbone benzophenone), #9 (2,2′-dihydroxybenzophenone), #38 (4-chloro-4′-hydroxybenzophenone), and #85 (decafluorobenzophenone) was further examined (**B**–**E**). For dose–response assays, compounds were tested at concentrations of 0–100 µg/mL. In addition, the antibiofilm activities of known positive controls, including eugenol, butoconazole, and ketoconazole, were evaluated in a dose-dependent manner (**F**–**H**). Eugenol was tested at 5–1000 µg/mL, whereas butoconazole and ketoconazole were tested at 5–100 µg/mL. Data are presented as mean ± standard deviation. Statistical significance was determined by one-way ANOVA followed by Tukey’s post hoc test. *, *p* < 0.05 compared with the untreated controls (N).

Screening of 101 benzophenone derivatives (15 non-halogenated derivatives, 36 mono-halogenated derivatives, 33 multi-halogenated derivatives, and 17 fluorine-rich derivatives) revealed differential antibiofilm activity against azole-resistant *C. albicans* DAY185 (Figure 1 and Table S1). The backbone benzophenone (#1) showed no inhibition, whereas several halogenated derivatives markedly suppressed biofilm development.

Notably, multiple halogen-substituted compounds reduced biofilm formation by more than 70%, with some derivatives such as 2-amino-5-bromobenzophenone and related bromo/fluoro-substituted analogues exhibiting strong inhibition, reducing biofilm levels to below ~30% of the untreated control (Figure 1 and Table S1). In contrast, most compounds had limited effects on planktonic cell growth (MIC ≥ 200 µg/mL in Table S1), indicating that the observed activity was primarily antibiofilm rather than fungicidal. These results demonstrate that halogenated benzophenones represent a distinct group with significant antibiofilm activity, and three active compounds (#9, #38, and #85) were chosen for further characterization.

### 2.2. Inhibition of Biofilm Formation by Decafluorobenzophenone and Visualization of Biofilm Architecture

Based on the primary screening, three active compounds (#9, #38, and #85) were further evaluated for dose-dependent inhibition of *C. albicans* biofilm formation (Figure 1A,C,D,E). The backbone benzophenone up to 100 µg/mL did not affect *C. albicans* biofilm formation (Figure 1B). Compounds #9 (2,2′-dihydroxybenzophenone) and #38 (4-chloro-4′-hydroxybenzophenone) showed moderate inhibition at 20 µg/mL and strong biofilm suppression at 50–100 µg/mL, indicating a clear concentration-dependent response. In contrast, #85 (decafluorobenzophenone) exhibited markedly higher potency, reducing biofilm formation to near-basal levels even at 10 µg/mL, with sustained inhibition at higher concentrations (Figure 1E). Additional dose–response analysis estimated the IC_50_ value of decafluorobenzophenone against *C. albicans* biofilm formation to be approximately 8.7 µg/mL (Figure S1). The antibiofilm activities of three hits were also investigated against the azole-sensitive *C. albicans* ATCC 10231. Three active compounds (#9, #38, and #85) at 20–50 µg/mL dose-dependently inhibited its biofilm formation while the backbone benzophenone (#1) inhibited only at 100 µg/mL (Figure S2). To compare the antibiofilm activity of the benzophenone derivatives investigated in this study with previously reported antifungal agents, eugenol [18], ketoconazole, and butoconazole [19] were additionally evaluated as positive controls. Eugenol showed minimal antibiofilm activity across the tested concentrations, whereas ketoconazole and butoconazole exhibited concentration-dependent inhibition of biofilm formation (Figure 1F–H). Among the tested compounds, decafluorobenzophenone demonstrated stronger antibiofilm activity at lower concentrations than the positive control compounds. In addition, the antibiofilm activity of decafluorobenzophenone was further evaluated against relevant *Candida* reference strains, *C. parapsilosis* ATCC 7330 and ATCC 22019 strains. Decafluorobenzophenone significantly inhibited biofilm formation in both *C. parapsilosis* strains in a concentration-dependent manner (Figure S3). Overall, #85 displayed the strongest antibiofilm activity among all tested compounds and was therefore selected as the lead candidate for subsequent mechanistic studies (Figure 1E).

**Figure 2:**
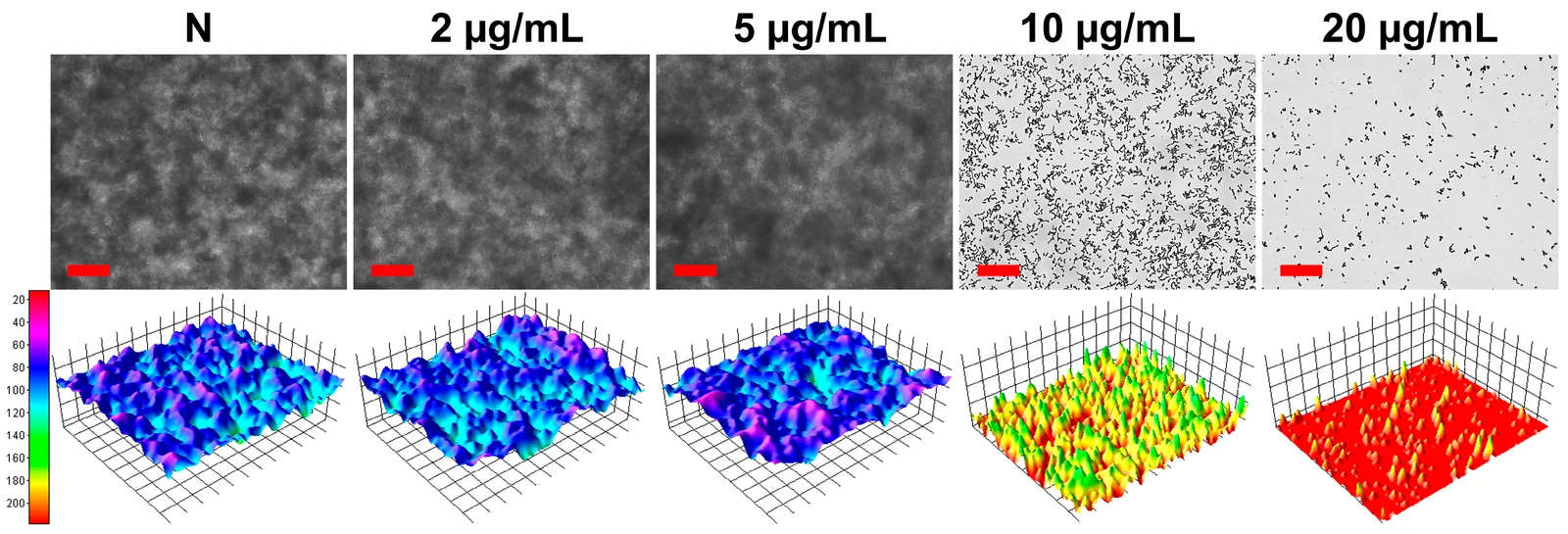
Microscopic observation of *C. albicans* biofilm inhibition. Two-dimensional and 3D images showing the biofilm architecture of *C. albicans* in the presence of decafluorobenzophenone. Images were obtained at concentrations of 0–20 µg/mL. Untreated controls (N). Red scale bar represents 100 μm.

The antibiofilm effect of decafluorobenzophenone (#85) was further visualized using 2D and 3D imaging (Figure 2). The untreated control formed dense, well-developed biofilms with extensive filamentous networks. At 2 and 5 µg/mL, only minor reductions in biofilm density were observed. In contrast, biofilm formation was markedly reduced from 10 µg/mL, with a clear decrease in biomass and structural integrity. At 20 µg/mL, only sparse and poorly organized cells remained. Three-dimensional images confirmed a substantial reduction in biofilm thickness and volume.

### 2.3. Suppression of Hyphal Transition and Biofilm-Associated Morphological Features by Decafluorobenzophenone

**Figure 3:**
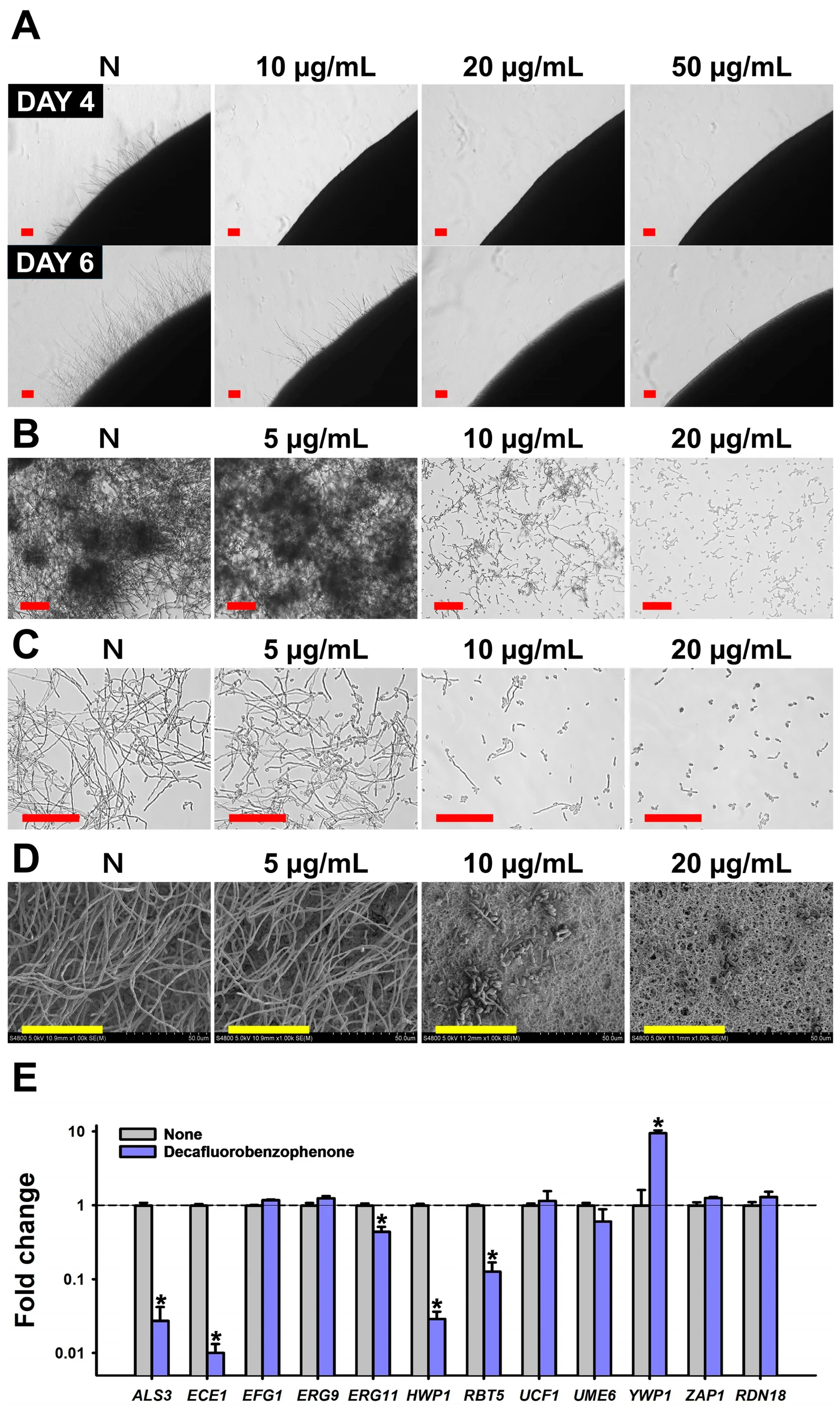
Inhibition of *C. albicans* hyphal transition by decafluorobenzophenone. Colony morphology (**A**), cell aggregation (**B**), germ tube and hyphal formation (**C**), hyphal structure by SEM (**D**), and relative expression levels of hyphae-related genes determined by qRT-PCR (**E**). For colony morphology analysis, cells were treated at concentrations of 0–50 µg/mL, and morphology was observed on days 4 and 6. For cell aggregation, germ tube and hyphal formation, and SEM analysis, cells were treated at concentrations of 0–20 µg/mL. Relative gene expression levels were analyzed by qRT-PCR and are presented as fold changes between decafluorobenzophenone-treated and untreated (N) cells. For qRT-PCR analysis, *C. albicans* cells were treated with decafluorobenzophenone at 10 µg/mL for 6 h under non-shaking condition. Relative expression levels of the target genes were normalized to *RDN18*, which was used as the internal housekeeping gene. * *p* < 0.05 compared with untreated controls. The experiment was independently performed twice. Untreated controls (N). Red scale bar represents 100 μm, whereas the yellow scale bar represents 50 μm.

The formation of filamentous hyphae from yeast cells is essential for *C. albicans* biofilm development and is a key virulence factor [20]. Hence, the effect of decafluorobenzophenone on hyphal transition and related morphological features was examined by observing colony morphology, cell aggregation, and filamentation (Figure 3). In Figure 3A, the untreated control exhibited typical wrinkled and filamentous colony morphology characteristic of hyphal growth. In contrast, colonies formed in the presence of decafluorobenzophenone at 10, 20, and 50 µg/mL appeared smoother, suggesting suppression of filamentous development. Figure 3B shows that control cells formed dense aggregates, consistent with active hyphal extension and cell–cell adhesion. Treatment with decafluorobenzophenone at 10 and 20 µg/mL markedly reduced cell aggregation, resulting in more dispersed and less clustered cell populations. In Figure 3C, microscopic observation revealed a strong inhibitory effect on hyphal formation. While the control cells developed elongated hyphae and filamentous networks, decafluorobenzophenone-treated cells predominantly remained in the yeast form. SEM analysis confirmed marked inhibition of hyphal transition (Figure 3D). The effect of decafluorobenzophenone on planktonic cell growth was evaluated at concentrations up to 400 µg/mL (Figure S4). The compound had minimal impact on cell proliferation, with no significant growth reduction even at 400 µg/mL. These findings suggest that decafluorobenzophenone primarily acts by suppressing hyphal transition and associated aggregation rather than inhibiting planktonic growth, which likely contributes to its strong antibiofilm activity.

### 2.4. Decafluorobenzophenone Modulates Hyphae- and Biofilm-Associated Gene Expression in C. albicans

To investigate the possible mechanism associated with the antibiofilm and anti-hyphal activities of decafluorobenzophenone, qRT-PCR analysis was performed using hyphae- and biofilm-related genes in *C. albicans* DAY185. Decafluorobenzophenone treatment significantly altered the expression levels of several virulence-associated genes involved in hyphal transition, adhesion, and biofilm formation (Figure 3E). In particular, the expression levels of *ALS3* (36-fold), *ECE1* (97-fold), *HWP1* (34-fold), *ERG11* (2.3-fold), and *RBT5* (7.7-fold) were markedly downregulated compared with untreated controls. Among these genes, *ECE1* (97-fold) exhibited the strongest reduction following treatment. Similar decreases in *ALS3*, *ECE1*, and *HWP1* expression have also been reported for other antibiofilm and anti-hyphal compounds in *C. albicans*. In contrast, *YWP1*, which encodes a yeast-form wall protein and is considered an indicator of hyphal inhibition, was upregulated (9.5-fold) following decafluorobenzophenone treatment [21]. These qRT-PCR findings suggest transcriptional-level support for decafluorobenzophenone treatment being associated with altered expression genes of hyphal transition and biofilm-associated genes, supporting its inhibitory effects on hyphal development and biofilm formation in *C. albicans *(Figure 3E).

### 2.5. Docking Analysis Suggests Possible Interactions with the Als3 Adhesion-Related Pocket

**Figure 4:**
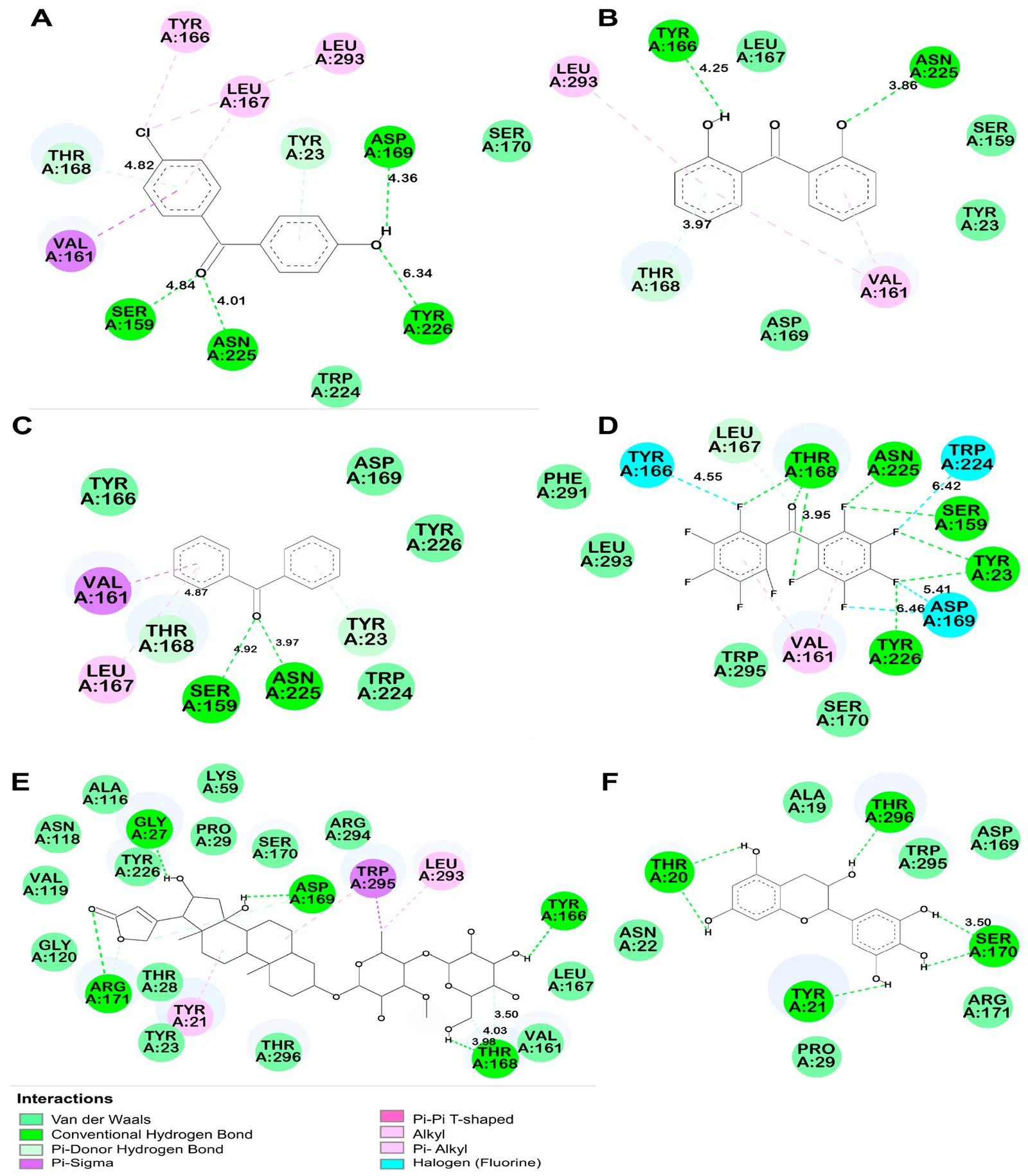
Molecular docking with Als3. Two-dimensional Als3 protein–ligand interactions of 4-chloro-4′-hydroxybenzophenone (**A**), 2,2′-dihydroxybenzophenone (**B**), benzophenone (**C**), decafluorobenzophenone (**D**), digitalin (**E**), and epigallocatechin (**F**). Digitalin and epigallocatechin were used as positive controls. Interaction types are color-coded as follows: light green (van der Waals contacts), dark green (conventional hydrogen bonds), pale green (π-donor hydrogen bonds), purple (π-sigma interactions), light pink (alkyl interactions), pink (π-alkyl interactions), magenta (π-π T-shaped interactions), and cyan (halogen/fluorine interactions).

The agglutinin-like sequence 3 (Als3) protein is essential for *Candida* cell attachment and biofilm development [22]. Therefore, molecular docking was utilized to predict the interactions between the benzophenone derivatives and the Als3 target cavity. Molecular docking simulation results predicted promising binding affinities compared to the standard positive controls (Table 1). Notably, compounds 4-chloro-4′-hydroxybenzophenone and 2,2′-dihydroxybenzophenone exhibited binding energies (−4.99 and −4.47 kcal/mol, respectively) comparable to positive controls like digitalin (−6.38 kcal/mol) and epigallocatechin (−4.90 kcal/mol) (Table 1). As shown in Figure 4, the lead compounds interacted closely with critical host–peptide binding residues as positive controls, including Tyr226, Val161, and particularly Thr168 and Ser170, which are recognized as key anchor residues for stabilizing high-affinity Als3 inhibitors [23,24], while 4-chloro-4′-hydroxybenzophenone and decafluorobenzophenone were found to interact within the vicinity of Ser170, a key residue for host–peptide binding (Figure S5A,B). The hydroxyl groups on the derivatives formed strong hydrogen bonds with Ser159, Tyr166, and Asn225 (Figure 4).

The parent compound, benzophenone (−4.31 kcal/mol), formed hydrogen bonds with Ser159 and Asn225 (Figure 4C and Table 1). Furthermore, the fluorine atoms in decafluorobenzophenone (−3.02 kcal/mol) created unique halogen bonds with Asp169 and Thr168 (Figure 4D and Table 1). Docking simulations suggest that the perfluorinated scaffold of decafluorobenzophenone may facilitate halogen-bonding interactions with residues within the Als3 pocket, including Thr168 (Figure 4D). These complexes were further stabilized by π-alkyl and π-sigma contacts between the aromatic rings and residues like Val161 and Leu293 (Figure 4). These findings suggest that benzophenone derivatives may adopt binding poses within the Als3 functional cavity that partially resemble those of known positive controls such as digitalin (Figure S6).

### 2.6. Decafluorobenzophenone Exhibits Low Toxicity Across Multiple Biological Models

**Figure 5:**
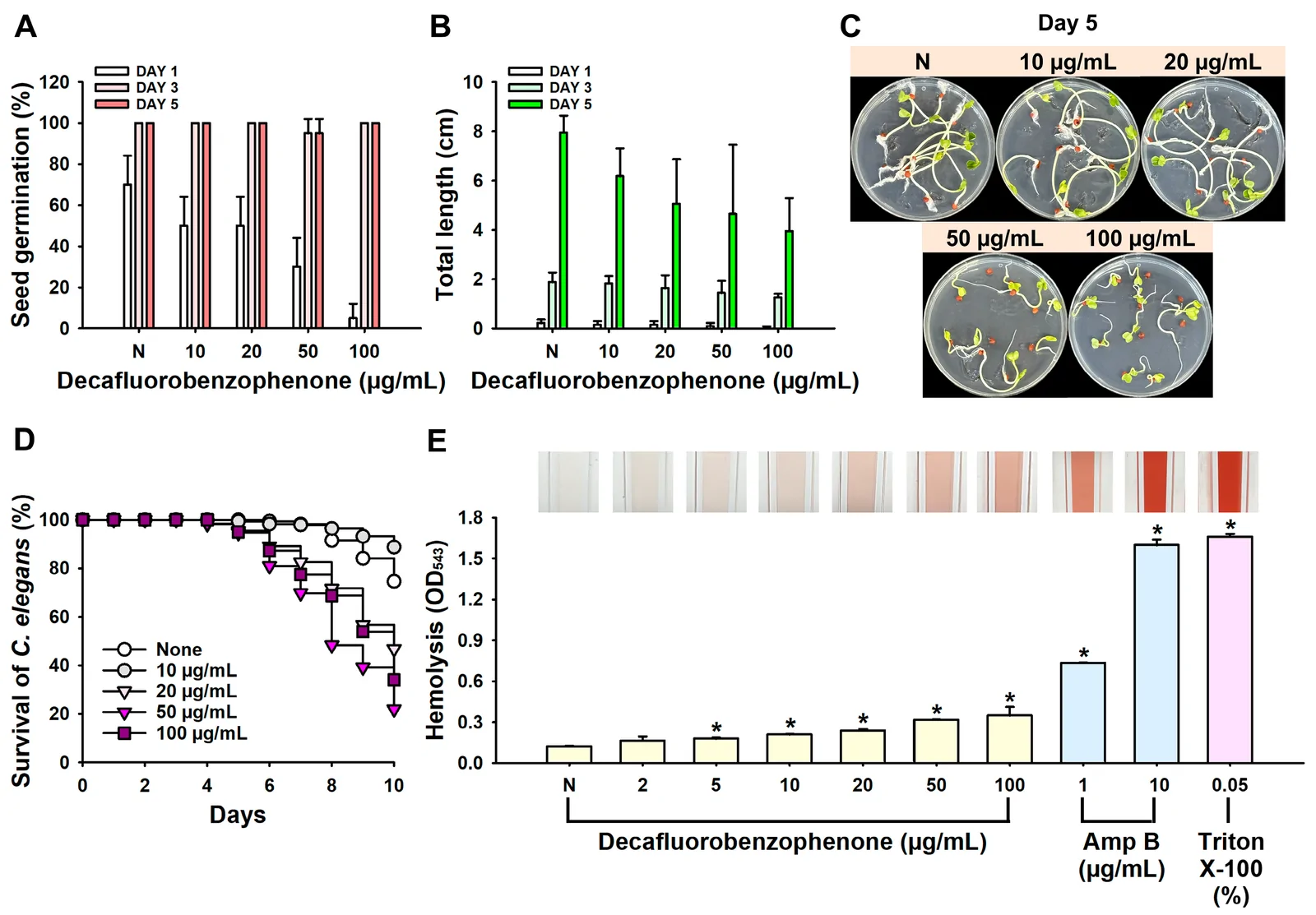
Toxicity analysis of decafluorobenzophenone. *Raphanus sativus* L. seed germination (**A**), total plant length (**B**), and representative images of seedlings after 5 days of growth (**C**). For toxicity assays using plant seed germination and nematode models, decafluorobenzophenone was tested at concentrations of 0–100 µg/mL; plant measurements were recorded on days 1, 3, and 5, while *C. elegans* survival was monitored daily for up to 10 days (**D**). Sheep red blood cell hemolysis (**E**). Amphotericin B (Amp B) and Triton X-100 were used as positive controls in the hemolysis assay. * *p* < 0.05 compared with the untreated control.

Based on previous studies, using *Raphanus sativus* L. seeds and *Caenorhabditis elegans* for chemical toxicity assessment [25,26], the potential toxicity of decafluorobenzophenone was evaluated by examining plant germination, overall growth, and *C. elegans* survival (Figure 5). Plant germination was not significantly affected by decafluorobenzophenone treatment up to 100 µg/mL for 5 days, indicating minimal impact on early developmental processes (Figure 5A). Also, total growth remained largely unchanged up to 10–20 µg/mL, with only a slight reduction observed at 50–100 µg/mL (Figure 5B,C). Similarly, *C. elegans* viability was not affected at 10 µg/mL; however, prolonged exposure to higher concentrations (20–100 µg/mL) resulted in a slight reduction in survival after 5 days, indicating low but concentration-dependent toxicity under extended treatment conditions (Figure 5D).

Also, hemolytic activity using sheep red blood cells was further investigated (Figure 5E); decafluorobenzophenone treatment up to 100 µg/mL slightly increased hemolysis in a dose-dependent manner while amphotericin B at 1 and 10 µg/mL significantly increased the hemolysis. Together, these results indicate that decafluorobenzophenone exhibits low toxicity toward non-target biological systems while maintaining strong antibiofilm activity.

In silico ADMET analysis was performed using the web-based prediction tools PreADMET, GUSAR, and SwissADME [27,28,29], through which the selected benzophenone derivatives were evaluated for predicted pharmacokinetic, drug-likeness, and toxicity-related properties (Table S2). Most compounds, including benzophenone, 4-chloro-4’-hydroxybenzophenone, and 2,2’-dihydroxybenzophenone, showed high predicted human intestinal absorption and favorable Caco-2/MDCK permeability, along with compliance with Lipinski, Ghose, Egan, and Veber rules. Decafluorobenzophenone exhibited high predicted intestinal absorption and permeability but showed increased lipophilicity (LogP 5.85) with one Lipinski violation and low predicted GI absorption, likely due to its extensive fluorination. Decafluorobenzophenone also demonstrated strong plasma protein binding and moderate hERG risk comparable to other derivatives. While Ames mutagenicity alerts were predicted for all compounds, carcinogenicity predictions were largely negative, except for a positive rat carcinogenicity alert for decafluorobenzophenone. Importantly, acute oral toxicity classifications placed most compounds in low-to-moderate toxicity categories (Class 4 or 5), consistent with the minimal toxicity observed in plant, nematode, and hemolysis assays.

### 2.7. Structure–Activity Relationship (SAR) Analysis of Benzophenone Derivatives as Antibiofilm Leads

**Figure 6:**
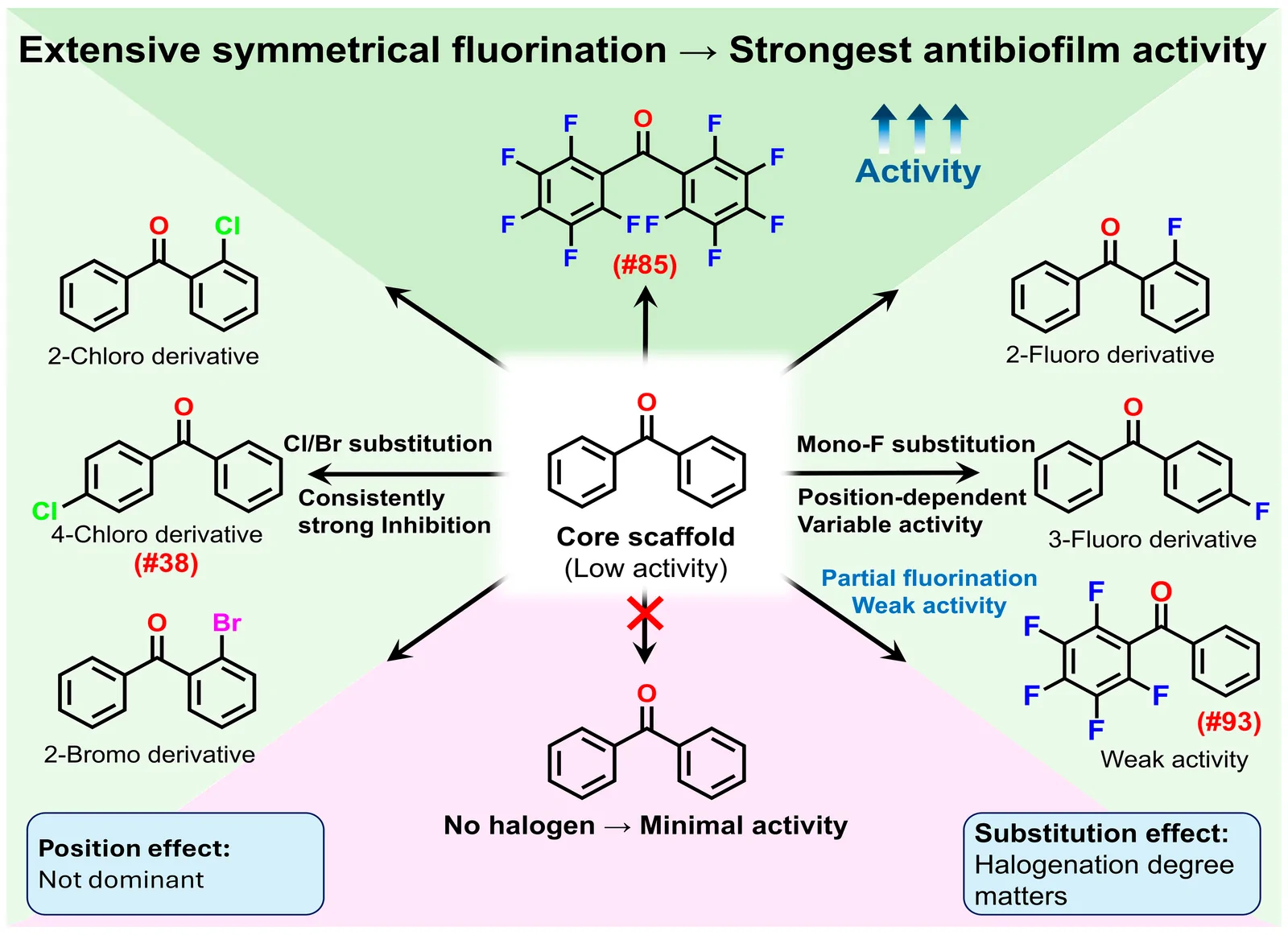
Structure–activity relationship of halogenated benzophenone derivatives. Representative structures illustrate the influence of halogen substitution on antibiofilm activity against azole-resistant *C. albicans*. Background colors indicate relative antibiofilm activity: dark green represents the strongest activity, light green represents lower or variable activity, and light pink represents minimal activity.

An SAR analysis for antibiofilm potency was performed based on the 50 µg/mL screening data (Figure 1 and Table S1). The parent benzophenone backbone showed no inhibition of biofilm formation, whereas most active compounds were halogenated derivatives, indicating that halogen substitution may contribute to antibiofilm activity in some benzophenone derivatives. Among the halogens, chloro- and bromo-substituted benzophenones consistently exhibited strong inhibition, frequently reducing biofilm formation to below 30% of the control (Figure 1 and Table S1). In particular, compounds bearing chloro groups, such as 4-chloro-4’-hydroxybenzophenone and other 2- or 4’-chloro derivatives, showed some of the most potent effects (Figure 6). Bromo-containing compounds also demonstrated pronounced activity, while fluoro-substituted derivatives showed variable effects depending on substitution patterns; however, the fully fluorinated compound, decafluorobenzophenone, displayed the strongest inhibition. In contrast, iodinated derivatives were generally less active. Positional effects were partially observed, as some ortho-substituted derivatives exhibited stronger activity than para-substituted analogues. However, the overall antibiofilm trend appeared to be more closely associated with the degree of halogenation than with substitution position alone. In particular, the fully fluorinated derivative decafluorobenzophenone (#85) exhibited the strongest antibiofilm activity, whereas compound #93, which contains only one perfluorinated phenyl ring, showed relatively weak activity. This comparison suggests that extensive and symmetrical fluorination across both aromatic rings, rather than partial fluorination on a single ring, may be important for enhancing antibiofilm activity, possibly through increased hydrophobic and halogen-bonding interactions. In contrast, partial fluorination may interfere with optimal molecular interactions and binding geometry. Since the present SAR analysis was primarily based on single-dose antibiofilm screening data, the observed activity trends should be interpreted with caution despite the additional IC_50_ evaluation of decafluorobenzophenone.

## 3. Discussion

This study demonstrates the antibiofilm activity of halogenated benzophenone derivatives against azole-resistant *C. albicans*. Among the screened library, several halogenated derivatives showed strong inhibition of biofilm formation without affecting planktonic cell growth. Notably, decafluorobenzophenone at 10 µg/mL displayed marked antibiofilm effects. The lead compound suppressed the hyphal transition while exhibiting minimal chemical toxicity.

Benzophenone derivatives have mainly been explored for their antimicrobial or antifungal growth-inhibitory activities, with halogen substitution known to enhance potency [9,11,14,15,30,31]. However, their effects on virulence-related processes have been less investigated. In previous studies, benzophenone-based derivatives were primarily evaluated for their direct antifungal effects on fungal growth [10], whereas the present study demonstrated that decafluorobenzophenone strongly inhibited biofilm formation at 10 µg/mL despite its much higher MIC (≥200 µg/mL). This distinction suggests that, beyond the previously reported antifungal properties of benzophenone derivatives, selected benzophenone scaffolds may also interfere with virulence-associated processes in *C. albicans.* In this study, screening of a benzophenone derivative library identified several halogenated benzophenones with antibiofilm activity, among which decafluorobenzophenone showed the strongest effect. Subsequent mechanistic analyses, performed primarily with decafluorobenzophenone, demonstrated that this compound selectively suppressed biofilm formation and hyphal transition in *C. albicans* without significantly affecting planktonic growth. This activity profile suggests a shift from antifungal growth inhibition to anti-virulence targeting, highlighting a new functional role for selected halogenated benzophenone derivatives, particularly decafluorobenzophenone, as potential antibiofilm and anti-hyphal lead candidates.

Previous studies have shown that halogen substitution can enhance the biological activity of small molecules by increasing lipophilicity, stability, and molecular interactions with biological targets [16]. For example, halogenated pyrrolopyrimidine derivatives specifically target *C. albicans* biofilm formation without affecting cell proliferation [17]. Compared with the halogenated pyrrolopyrimidine derivatives reported previously [17], decafluorobenzophenone exhibited relatively weaker antifungal growth-inhibitory activity but showed potent antibiofilm activity at low concentrations, with minimal effects on planktonic growth. These differences suggest that the present compound may act more selectively on virulence-associated processes rather than directly inhibiting fungal proliferation. Consistent with these reports, the present study revealed that halogen substitution may contribute to the antibiofilm activity of some benzophenone derivatives. Compounds containing chloro- and bromo-substituents frequently showed strong inhibition, while mono-fluoro-substitutions displayed variable effects depending on substitution patterns. However, the overall activity pattern was not fully explained by the degree, type, or symmetry of halogenation alone. Although decafluorobenzophenone showed the strongest antibiofilm activity, other active compounds, such as 4-chloro-4′-hydroxybenzophenone and 2,2′-dihydroxybenzophenone, indicate that additional structural features may also contribute to activity. These observations suggest that antibiofilm activity may reflect a combination of multiple factors, including substitution position, hydrophobicity/lipophilicity, electronic effects, steric arrangement, hydrogen-bonding capacity, and overall substitution pattern. Therefore, the present SAR analysis should be interpreted as exploratory and qualitative. Further quantitative analyses using systematically designed analogue series, such as principal component analysis or regression-based modeling, will be needed in future studies to more precisely define the relationships between structural features and antibiofilm activity.

Positional effects were also evident, as several active compounds contained ortho-halogen substitutions, indicating that the spatial orientation of substituents may influence antibiofilm efficacy. Furthermore, several multi-halogenated derivatives showed stronger inhibition than mono-substituted analogues, suggesting that increased halogenation may contribute to activity in certain structural contexts. A similar trend has been reported for halogenated pyrrolopyrimidine derivatives against *C. albicans* [17].

Fluorinated derivatives were more prevalent in the compound library, reflecting the common use of fluorine substitution to enhance lipophilicity and molecular stability. In this study, extensive fluorination, particularly in decafluorobenzophenone, was associated with the strongest antibiofilm activity, while chloro- and bromo-substituted compounds also showed notable effects depending on substitution patterns. These findings suggest that the type and number of halogen atoms influence activity. Further studies exploring multi-halogenated derivatives containing combinations of Br, Cl, and I at different positions may help to further improve antibiofilm activity.

Microscopic analyses further revealed that decafluorobenzophenone strongly disrupted biofilm architecture and inhibited hyphal development. Since filamentous hyphae play a central role in adhesion, invasion, and biofilm maturation, suppression of the yeast-to-hypha transition likely represents a major mechanism underlying the observed antibiofilm activity. The reduction in colony wrinkling, cell aggregation, and filament formation suggests that decafluorobenzophenone interferes with virulence-associated processes rather than directly inhibiting cell growth. This interpretation is supported by the observation that planktonic growth was largely unaffected even at high concentrations, indicating a predominantly anti-virulence mode of action.

qRT-PCR analysis additionally supported the inhibitory effects of decafluorobenzophenone on hyphal transition and biofilm-associated virulence pathways in *C. albicans*. Treatment with decafluorobenzophenone reduced the expression levels of several genes associated with adhesion, filamentation, and biofilm maturation, including *ALS3*, *ECE1*, *HWP1*, *ERG11*, and *RBT5*, while *YWP1* expression was increased compared with untreated controls. Since *ALS3*, *ECE1*, and *HWP1* are closely involved in hyphal development and host adhesion, their transcriptional repression may contribute to the reduced filament formation and weakened biofilm structure observed microscopically. More specifically, *ALS3* and *HWP1* function as hypha-associated adhesins that contribute to cell attachment and biofilm establishment, whereas *ECE1* is closely associated with hyphal growth and virulence; therefore, their downregulation is consistent with the observed reduction in cell aggregation and filamentous growth. *ERG11* is involved in ergosterol biosynthesis, while *RBT5* contributes to heme-iron acquisition; however, their relatively modest downregulation compared with *ALS3*, *ECE1*, and *HWP1* suggests only limited transcriptional effects on sterol- and iron-associated cellular responses. In addition, *YWP1* has been reported to reduce cell adhesion and is associated with the yeast-form state in *C. albicans*, and thus, its increased expression may further contribute to the inhibition of hyphal transition and biofilm formation by decafluorobenzophenone [32]. Similar regulatory patterns have been reported for other antibiofilm and anti-hyphal compounds targeting virulence-associated pathways in *C. albicans* [4,21]. Therefore, these findings suggest transcriptional-level support that decafluorobenzophenone treatment is associated with altered expression of hyphae- and biofilm-associated genes, consistent with the observed inhibition of hyphal development and biofilm formation. Additionally, the coordinated repression of *ALS3*, *ECE1*, and *HWP1* suggests that the anti-hyphal effects of decafluorobenzophenone may also involve broader changes in filamentation-associated pathways, in addition to the possible involvement of *Als3*-related adhesion. The cAMP-PKA pathway involving Tpk1/Tpk2 and the Cek1-mediated MAPK pathway are established regulators of hyphal morphogenesis in *C. albicans* [33,34], and modulation of these or related upstream signaling pathways may represent an additional possibility.

The molecular docking protocol was validated using specific positive controls previously reported to inhibit Als3 [35,36]. Although decafluorobenzophenone showed a weaker predicted docking score (−3.02 kcal/mol) than several other derivatives, docking scores may not directly reflect experimental activity [37]. Moreover, molecular interactions in aqueous environments can be influenced by hydrophobic effects and surrounding hydration water [38]. Therefore, the strong antibiofilm activity of decafluorobenzophenone may reflect additional physicochemical and cellular factors beyond its predicted affinity for Als3, including potential differences in cell-wall or membrane penetration, cellular availability, and possible multi-target effects. Docking analysis predicted that decafluorobenzophenone may adopt an interaction pattern partially overlapping with that of digitalin, involving residues such as Asp169 and Thr168 while bridging the hydrophobic cleft through contacts with Val161 and Leu293, while remaining positioned within the Ser170 binding vicinity. Similarly, plant-derived aromatic compounds such as eugenol and cinnamaldehyde, as well as the plant indole alkaloid globospiramine, have been reported to inhibit biofilm formation by disrupting adhesion mechanisms and targeting Als3 [23,39]. While these computational insights provide preliminary support for possible interactions between decafluorobenzophenone and the Als3 peptide-binding cavity and suggest the potential involvement of benzophenone derivatives in Als3-related adhesion, they should be interpreted as supportive rather than definitive evidence. Protein kinases contain conserved HRD and DFG motifs in which aspartate residues contribute to catalytic activity and ATP coordination, respectively [40]. Given the predicted interaction of decafluorobenzophenone with Asp169 in the Als3 docking model, possible interactions with aspartate-containing kinase environments may be considered as a working hypothesis; however, this possibility remains speculative and requires direct evaluation of kinase activity and phosphorylation events. Further target-validation studies, such as biochemical binding assays and Als3 mutant analysis, may help clarify the possible contribution of Als3-related adhesion to the observed activity.

Importantly, toxicity analyses indicated that decafluorobenzophenone has a favorable safety profile at active concentrations. The compound showed minimal effects on seed germination, plant growth, nematode, and erythrocyte hemolysis. In *C. elegans*, no toxicity was observed at 10 µg/mL, while only a slight reduction in survival was detected following prolonged exposure at higher concentrations (20–100 µg/mL). Also, ADMET predictions suggest that halogenated benzophenones possess acceptable drug-likeness profiles, although the high lipophilicity and predicted mutagenicity alerts of decafluorobenzophenone warrant further experimental toxicological validation. These results suggest relatively low biological toxicity and support its potential as a safe antibiofilm candidate.

Although drug-resistant clinical *C. albicans* isolates were not included in the present study, decafluorobenzophenone demonstrated antibiofilm activity against the azole-resistant *C. albicans* strain DAY185 and the azole-sensitive *C. albicans* ATCC 10231, as well as relevant *Candida* reference strains, including *C. parapsilosis* ATCC 7330 and ATCC 22019. Thus, the antibiofilm activity of decafluorobenzophenone was not restricted to the azole-resistant *C. albicans* DAY185 background and was also observed in a non-albicans *Candida* species. Similar cross-strain and cross-species activity has been reported for 2-aryloxazoline derivatives, which remained active against *Candida* clinical isolates with reduced susceptibility or resistance to conventional antifungals and inhibited biofilm formation and hyphal morphogenesis in both *C. albicans* and *C. tropicalis* [41]. However, these derivatives also showed strong growth-inhibitory activity, whereas decafluorobenzophenone exerted potent antibiofilm activity with relatively weak effects on planktonic growth, indicating a distinct activity profile. Importantly, previous studies have also demonstrated substantial species- and strain-dependent differences in the susceptibility of *Candida* biofilms to antifungal agents [42], emphasizing the value of evaluating antibiofilm compounds across different *Candida* species and strain backgrounds. These findings support its potential broad-spectrum antibiofilm activity and provide a strong rationale for future evaluation using a wider range of clinical drug-resistant *C. albicans* isolates. Although antibiofilm activity was also observed in azole-sensitive *C. albicans* ATCC 10231 and *C. parapsilosis* reference strains, the number of additional strains and species examined in this study was limited. Therefore, evaluation across a broader range of *C. albicans* strains and non-albicans *Candida* species would help determine the consistency of these findings across diverse *Candida* backgrounds.

This study highlights the potential of selected benzophenone derivatives, particularly decafluorobenzophenone, as antibiofilm lead candidates. Further studies focused on target validation, clinical drug-resistant *C. albicans* isolates, and in vivo infection models will be essential to better understand its mechanism of action and therapeutic potential.

## 4. Materials and Methods

### 4.1. Strains, Chemicals, and Culture Conditions

*C. albicans* strains DAY185 (azole-resistant: MIC > 1000 μg/mL for fluconazole, itraconazole, and posaconazole) and ATCC 10231 (azole-sensitive) and *Candida parapsilosis* strains ATCC 7330 and ATCC 22019 were obtained from the American Type Culture Collection (ATCC, Manassas, VA, USA). Strains preserved in glycerol stocks were streaked onto solid potato dextrose agar (PDA) plates only for recovery and isolation of fresh colonies, as previously described [43,44]. *C. albicans* strains were subsequently cultured in liquid potato dextrose broth (PDB), which was used as the culture and biofilm assay medium, consistent with its use in a recent study of *C. albicans* biofilm formation [45]. *C. parapsilosis* strains were cultured in YM medium supplemented with 2% glucose for subsequent experiments. At 37 °C, cultures in liquid medium were cultivated with agitation adjusted to 250 rpm. The 101 benzophenone derivatives (Table S1), eugenol, ketoconazole, butoconazole and amphotericin B (Amp B), were purchased from Combi-Blocks (San Diego, CA, USA) and Sigma-Aldrich (St. Louis, MO, USA) and subsequently solubilized in dimethyl sulfoxide (DMSO). Purity values for the tested benzophenone derivatives are summarized in Table S1 where available. Among the 101 benzophenone derivatives, decafluorobenzophenone was selected for subsequent assays based on the initial antibiofilm screening. The concentration ranges used in these assays were selected according to the experimental objective, with lower concentrations used for follow-up biofilm, morphological, and gene-expression analyses, whereas broader concentration ranges were used for dose–response and toxicity evaluations. The final DMSO content was kept under 0.1% (ν/ν) throughout the experiments and was confirmed to have no significant effect on *C. albicans* growth or biofilm production. Amphotericin B, eugenol, ketoconazole, and butoconazole were employed as positive control.

### 4.2. Minimum Inhibitory Concentration (MIC) Assay

Planktonic growth inhibition was evaluated using a custom high-inoculum assay. Overnight cultures were initiated from a freshly isolated colony of *C. albicans* DAY185 by transferring the colony using a 250 mL Erlenmeyer vessel charged with 25 mL PDB and maintained at 37 °C on a rotary shaker. The overnight culture was diluted 1:50 with fresh PDB medium to obtain an initial inoculum of approximately 1 × 10^6^ cells/mL. The diluted culture received a benzophenone derivative (total 101 compounds) to reach at final concentration ranging from 10 to 200 µg/mL, and then the samples were transferred to 96-well assay plates. Following incubation for 24 h at 37 °C under agitation, optical density at 600 nm (OD_600_) was recorded with a Multiskan SkyHigh microplate reader (Thermo Fisher Scientific, Waltham, MA, USA) [46] to assess planktonic cell growth. The growth-inhibitory endpoint was defined as the lowest tested concentration that prevented detectable fungal proliferation.

### 4.3. Biofilm Inhibition Assay Using Crystal Violet

Biofilm inhibition assay was achieved in 96-well polystyrene plates (SPL Life Sciences, Pocheon, Republic of Korea) based on an established protocol [32]. First, overnight cultures for 48 h of *C. albicans* were diluted into fresh PDB medium using dilution at a 1:50 ratio, resulting in a final density of ~10^6^ cells/mL. Benzophenone derivatives were added (0, 10, 20, 50, and 100 µg/mL), and the cultures were dispensed into 96-well plates and incubated at 37 °C for 24 h without agitation. The four benzophenone compounds evaluated in this assay—benzophenone, 2,2′-dihydroxybenzophenone, 4-chloro-4′-hydroxybenzophenone, and decafluorobenzophenone—were dissolved in DMSO. The final DMSO concentration in all compound-treated groups was maintained below 0.1% (*v*/*v*). The planktonic cells were discarded, and then the wells were rinsed three times with distilled water. To stain the remaining cells in each well, 0.1% crystal violet solution (w/ν, Sigma-Aldrich) [47] was added to the 96-well plate and incubated for 20 min, and the crystal violet solution was discarded and washed as previously described. After removal of the solution, the bound dye was solubilized with 95% ethanol [21]. Absorbance was measured at 570 nm using a Multiskan SkyHigh microplate reader. Biofilm experiments were performed independently at least three times.

### 4.4. Live Imaging Microscopy Assay for Biofilm Formation

To evaluate the antibiofilm activity of decafluorobenzophenone against *C. albicans* DAY185, biofilms were formed according to a previously reported method [21] and treated with decafluorobenzophenone at final concentrations (0, 2, 5, 10, and 20 µg/mL). After discarding the planktonic cells, the wells were rinsed with distilled water. To enhance visualization of the attached biofilm cells, 0.1% crystal violet was added and stained for 20 min. The crystal violet solution was discarded and washed three times with distilled water. The residual liquid was carefully removed to avoid detaching the biofilm cells before observation. The biofilm cells were moistened with 30 μL of phosphate-buffered saline (PBS) and visualized using live cell imaging microscopy, iRiS™ Digital Cell Imaging System (Logos BioSystems, Anyang, Republic of Korea). The biofilm cells were observed under a microscope and captured as images. The acquired images were subsequently processed using ImageJ (version 1.54g; https://imagej.net/ij; accessed on 4 November 2025) software to generate color-rendered two-dimensional (2D) and three-dimensional (3D) reconstructions [32]. For each concentration, six replicates were analyzed, and the experiment was performed independently twice.

### 4.5. Hyphal Formation and Cell Aggregation by Live Imaging Microscopy

*C. albicans* DAY185 cells were re-inoculated into 10 mL of PDB medium at a 1:50 dilution to obtain a final cell density of 10^6^ CFU/mL. The re-inoculated culture was aliquoted into 1.7 mL tubes with or without decafluorobenzophenone (5, 10, and 20 µg/mL). Before the incubation, all tubes were vortexed thoroughly and incubated without agitation for 24 h at 37 °C [4]. After incubation, the cell culture was gently tapped and carefully transferred to 100 mm Petri dishes for bright-field microscopic observation using the iRiS™ Digital Cell Imaging System (Logos Biosystems) at 10× and 20×. To investigate *C. albicans* colony morphology on a PDA (Potato dextrose agar) plate, 48 h overnight cultures were dropped on PDA plates containing decafluorobenzophenone (10, 20, and 50 µg/mL) and incubated for 6 days at 37 °C without agitation. Colony morphologies were observed on an optical microscope (iRiS™ Digital Cell Imaging System). At least two independent experiments were conducted.

### 4.6. SEM Imaging Assay for Biofilm Inhibition

Scanning electron microscopy (SEM) was performed to analyze hyphal development as previously described [48]. Prior to use, nylon membranes (0.45 μm pore size; Whatman, Maidstone, UK) were cut into approximately 0.4 cm × 0.4 cm pieces on aluminum foil, placed in a beaker, autoclaved for sterilization, and dried in a drying oven at 60 °C for at least 24 h. Following the same procedure as the biofilm assay, the 1:50 re-inoculated overnight culture was mixed with decafluorobenzophenone at final concentrations (5, 10, and 20µg/mL). Mixed cultures were dispensed into each well, and one sterilized nylon membrane was placed into each well using sterile forceps. A plate was incubated at 37 °C for 24 h without agitation. The biofilms formed on the membranes were fixed using a fixing solution composed of 2.5% glutaraldehyde and 2.5% formaldehyde at 4 °C for 24 h. Samples were dehydrated in a graded ethanol series from 50% to 99%, with each step performed for 20 min and repeated three times. Following critical-point drying using an HCP-2-unit (Hitachi, Tokyo, Japan), the samples were platinum sputter-coated and examined using an S-4800 SEM (scanning electron microscope, Hitachi, Tokyo, Japan); an accelerating voltage of 5.0 kV was applied, and images were obtained at 1000× magnification [49].

### 4.7. RNA Isolation and Quantitative Real-Time PCR

For RNA isolation of *C. albicans* DAY185, 48 h overnight culture of *C. albicans* was re-inoculated into fresh PDB medium at a 1:50 dilution to obtain an OD_600_ value of approximately 0.1. For the untreated control (None), 25 mL of culture was re-inoculated, whereas 25 mL cultures were prepared for the decafluorobenzophenone-treated samples. Decafluorobenzophenone was added to the culture at a final concentration of 10 µg/mL. A concentration of 10 µg/mL was selected because it showed strong antibiofilm activity with minimal effects on planktonic growth. The cultures were then incubated at 37 °C for 6 h under non-shaking conditions. After incubation, RNAlater solution (RNA stabilization solution, Thermo Fisher Scientific) was added to the cell cultures to minimize RNA degradation, and the cultures were mixed thoroughly prior to harvesting. Total RNA was isolated and purified using a Qiagen RNeasy Mini Kit (Valencia, CA, USA). Following RNA isolation, RNA concentration and purity were measured using a NanoVue UV–Vis spectrophotometer (GE Healthcare, Chicago, IL, USA) [50].

qRT-PCR analysis was performed using gene-specific primers to evaluate the expression levels of genes associated with hyphal transition and biofilm formation (*ALS3*, *ECE1*, *EFG1*, *ERG9*, *ERG11*, *HWP1*, *RBT5*, *UCF1*, *UME6*, *YWP1*, *ZAP1*, *RDN18*). The relative expression levels of the target genes were normalized to *RDN18* (Table S3), which was used as the internal housekeeping gene. qRT-PCR analysis was carried out using M-MLV reverse transcriptase (Thermo Fisher Scientific, Waltham, MA, USA), an RNase inhibitor (Thermo Fisher Scientific), SYBR Green real-time PCR master mix (Thermo Fisher Scientific), and a StepOne Real-Time PCR System (Applied Biosystems, Foster City, CA, USA). Each experiment was conducted with at least two independent colonies [50].

### 4.8. Molecular Docking Analysis with Agglutinin-like Sequence 3 (Als3) Protein

The high-resolution structure of the Als3 protein of *C. albicans* (1.4 Å) was accessed under PDB ID 4LEB from the RCSB Protein Data Bank (https://www.rcsb.org/; accessed 19 February 2026) [51]. This model features a co-crystallized hepta-threonine peptide designated as chain B. Preparation of the ligands involved converting their SMILES strings into 3D SDF files using DataWarrior v5.5.0 (Allschwil, Switzerland) (accessed 19 February 2026) [52]. Molecular docking simulations were performed via the Pharmaco-Net platform (https://app.pharmaco-net.org/; accessed 19 February 2026), consistent with the parameters previously reported by our and other groups for modeling Als3 adhesin [36,53]. The 4LEB structure and ligand files were uploaded to the Pharmaco-Net server. The hepta-threonine peptide (chain B) was removed using the Pocket Finder module. The remaining protein structure was repaired to replace missing residues and optimize its energy. The docking grid was centered on the key interacting residues within the N-terminal peptide-binding cavity of Als3 (chain A). The docking box was defined as a 155-unit cube with central coordinates of X: 1.8, Y: 5.0, and Z: −13.3. The presence of the binding pocket within these grid coordinates was verified using the CASTp server (http://sts.bioe.uic.edu/castp/index.html?2was; accessed 19 February 2026) [54]. Docking simulations were performed with the AI-Dock Plus and DeepCalici Plus modules, which calculated the binding energies and affinities of ligands docked to the defined grid. Each compound was docked across five independent runs, yielding consistent results (Figure S4). Discovery Studio Visualizer (v25.1.0, Dassault Systèmes, Vélizy-Villacoublay, France, accessed 19 February 2026) was then used to visualize the resulting protein–ligand interactions and docked poses.

### 4.9. Toxicity Assay: Plant Seed Germination and Nematode Models

Plant seed germination and *C. elegans* survival assays have been previously employed for toxicity assessment [17]. In the present study, the experimental conditions for the plant toxicity assay were empirically optimized to obtain consistent germination and growth. Radish seeds were initially washed five times with distilled water that had been pre-warmed at 60 °C for 5 h in a drying oven. The seeds were then soaked in warm distilled water for 3 h, followed by three additional washes with distilled water and drying for 6 h at room temperature. After drying, seeds of uniform size and shape were selected, and ten seeds were placed on each Murashige and Skoog (MS) plate using sterilized forceps, which were flame-sterilized before each use. MS plates were prepared by supplementing 0.86 g/L MS medium with 0.7% agar, pouring 50 mL of the medium into a 250 mL flask, and sterilizing by autoclaving. For treatment plates, the autoclaved medium was allowed to cool with stirring, after which decafluorobenzophenone was added to achieve final concentrations of 10, 20, 50, and 100 µg/mL. Seed plates were incubated at room temperature for 5 days. Seed germination and total length were measured and recorded on days 1, 3, and 5.

The *C. elegans* fer-15(b26); fem-1(hc17) strain was used to assess the toxicity of decafluorobenzophenone in a nematode model, following the method described [21]. *C. elegans* was used as a preliminary whole-organism toxicity model to evaluate the potential in vivo toxicity of benzophenone derivatives in a simple and cost-effective system. The *C. elegans* survival assay was performed following a previously described method with modifications [32]. *C. elegans* were synchronized by bleaching gravid adults, and the collected eggs were cultured on NGM plates seeded with *Escherichia coli* OP50 until adulthood. Prior to the experiment, stage-synchronized adult *C. elegans* were collected and washed 3–4 times with M9 buffer to remove residual bacteria. Approximately 30 nematodes were added to each well of a 96-well plate containing 200 µL of M9 buffer with decafluorobenzophenone (10, 20, 50, and 100 µg/mL). The control group was treated with M9 buffer only. The 96-well plates were sealed with wrap and incubated at 25 °C without shaking, and survival was monitored for up to 10 days. Survival was recorded daily throughout the experimental period. The result of nematode survival was presented as a percentage. All experiments were independently performed twice.

### 4.10. Toxicity Assay: Sheep Erythrocyte Hemolysis Assay

The general sheep erythrocyte hemolysis procedure was adapted from a previously reported method [55]. In the present study, the microbial culture step used in the previous assay was omitted, and sheep red blood cells (RBCs, MB Cell, Seoul, Republic of Korea) were directly exposed to decafluorobenzophenone to evaluate compound-induced hemolysis. Prior to the experiment, RBCs were processed by using centrifugation for 5 min at 3000 rpm to precipitate the erythrocytes [23]. For washing the erythrocytes, supernatant was eliminated and phosphate-buffered saline (PBS) was utilized for washing. The washing step was repeated until the supernatant became clear. After washing, the RBC pellet was resuspended in PBS, and then the washed RBC suspension was diluted into PBS to obtain a final RBC concentration of 3.33% (ν/ν). Subsequently, the diluted solution was aliquoted into 14 mL tubes, and amphotericin B (1 and 10 µg/mL), decafluorobenzophenone (2, 5, 10, 20, 50, and 100 µg/mL), and Triton X-100 (0.05%) were added at final concentrations. Triton X-100 was used as a positive control. The mixtures were incubated at 37 °C for 3 h in shaking incubator. Following this period, each sample was moved to 1.5 mL microtubes and centrifuged at 8000 rpm for 10 min to separate intact RBCs. The supernatants were transferred to a cuvette, and absorbance at 543 nm (OD_543_) was measured using a Multiskan SkyHigh microplate reader.

### 4.11. In Silico Analysis of ADME and Toxicity Profiles of Selected Benzophenone Derivatives

The absorption, distribution, metabolism, excretion, toxicity, and drug-likeness of the benzophenone derivatives #1 (benzophenone), #38 (4-chloro-4’-hydroxybenzophenone), #9 (2,2’-dihydroxybenzophenone), and #85 (decafluorobenzophenone) were predicted using online in silico tools including PreADMET (https://preadmet.webservice.bmdrc.org/; accessed on 20 November 2025), GUSAR (http://www.way2drug.com/gusar/; accessed on 20 November 2025), and SwissADME (https://www.swissadme.ch/; accessed on 20 November 2025), which were accessed on 20 November 2025. According to Lipinski’s rule of five, compounds intended for oral administration are generally characterized by a molecular weight not exceeding 500 g/mol, LogP not exceeding 5, up to five hydrogen bond donors, up to ten hydrogen bond acceptors, and a polar surface area within 140 Å^2^ [17].

### 4.12. Structural Classification and Structure–Activity Relationship (SAR) Analysis of Benzophenone Derivatives

Structure–activity relationship (SAR) analysis was carried out using the results obtained from the primary antibiofilm screening performed at 50 μg/mL (Table S1). Compounds were categorized as active (≥70% inhibition), moderately active (40–70%), or inactive (<40%) depending on remaining biofilm biomass compared with untreated controls. Dose–dependent experiments were thereafter performed with compounds that exhibited strong activity in the initial screening. Then, structural features of derivatives were assessed to examine the halogen atom (F, Cl, Br, or I), the number of substitutions (mono-, di-, and multi-substituted), and the position of substitution on the aromatic ring (ortho, meta, or para). The correlation between halogen atom characteristics and antibiofilm activity was assessed using structural parameters. In addition, representative molecular structures were selected to illustrate structural features related to activity and to help identify potential lead compounds for subsequent mechanistic studies.

### 4.13. Statistical Analysis

Data are mainly expressed as mean values including standard deviations (SD). In all experiments, statistical comparisons of treated groups and untreated groups were performed using Student’s *t*-test. In addition, for experiments including multiple group comparisons, statistical analysis was additionally carried out by one-way analysis of variance (ANOVA) followed by Tukey’s post hoc test using GraphPad Prism version 11.0 (GraphPad Software, San Diego, CA, USA). Results were defined as significant at a *p*-value of ≤0.05. Figures were generated with SigmaPlot version 14.0 (Systat Software, San Jose, CA, USA).

## 5. Conclusions

This study demonstrates that halogenated benzophenone derivatives, particularly decafluorobenzophenone, effectively inhibit biofilm formation and hyphal transition in azole-resistant *C. albicans*. The compound showed strong antibiofilm activity with minimal effects on planktonic growth and low toxicity in biological models. These findings highlight the potential of selected benzophenone derivatives, particularly decafluorobenzophenone, as anti-virulence lead candidates for controlling biofilm-associated fungal infections.

## Figures and Tables

**Table 1:** Predicted binding energies (kcal/mol) and affinities (µM) of benzophenone derivatives and positive controls (digitalin, epigallocatechin, diazepam, and rutin) against the Als3 protein.

Name	Predicted Binding Energy (kcal/mol)	Predicted Binding Affinity (µM)
Digitalin	−6.38	0.024
4-chloro-4′-hydroxybenzophenone	−4.99	0.0686
Epigallocatechin	−4.90	0.0827
2,2′-dihydroxybenzophenone	−4.47	0.1206
Diazepam	−5.57	0.2585
Benzophenone	−4.31	1.5556
Rutin	−5.90	1.6883
Decafluorobenzophenone	−3.02	1.8373

## Data Availability

The data utilized in this study can be found within the main text of the article and the accompanying Supplementary Materials.

## Supplementary Materials

The following supporting information can be downloaded at: https://www.mdpi.com/article/10.3390/ijms27177528/s1.

## Abbreviations

The following abbreviations are used in this manuscript:

*C. albicans*	*Candida albicans*
*C. parapsilosis*	*Candida parapsilosis*
*C. elegans*	*Caenorhabditis elegans*
ATCC	American Type Culture Collection
Amp B	Amphotericin B
MIC	Minimum inhibitory concentration
PDA	Potato dextrose agar
PDB	Potato dextrose broth
DMSO	Dimethyl sulfoxide
OD	Optical density
CFU	Colony-forming unit
PBS	Phosphate-buffered saline
SEM	Scanning electron microscopy
qRT-PCR	Quantitative real-time polymerase chain reaction
Als3	Agglutinin-like sequence 3
MS	Murashige and Skoog
RBC	Red blood cell
ADMET	Absorption, distribution, metabolism, excretion, and toxicity
IC_50_	Half-maximal inhibitory concentration
SAR	Structure–activity relationship
SD	Standard deviation
