# Supplementary material for: Antibiofilm and Anti-Hyphal Activities of Halogenated Benzophenones Against Azole-Resistant Candida albicans
Source: Int J Mol Sci. 2026 Aug 22;27(17):7528. doi: 10.3390/ijms27177528 (PMC13566032; doi:10.3390/ijms27177528)
Supplement: Supplementary file 1 [file ijms-27-07528-s001.zip › ijms-4465257-supplementary.pdf]

Article

Antibiofilm and Anti-Hyphal Activities of Halogenated Benzophenones Against Azole-Resistant *Candida albicans*

Juyeon Jo [1], Ziyad Abdelaal [1], Yong-Guy Kim [2], Jin-Hyung Lee [2,*] and Jintae Lee [1,*]

[1] School of Chemical Engineering, Yeungnam University, 280 Daehak-Ro, Gyeongsan 38541, Republic of Korea; dusdl6631@yu.ac.kr (J.J.); zabdelaal@yu.ac.kr (Z.A.)

[2] Institute of Clean Technology, Yeungnam University, 280 Daehak-Ro, Gyeongsan 38541, Republic of Korea; yongguy7@ynu.ac.kr

* Correspondence: jinhlee@ynu.ac.kr (J.-H.L.); jtlee@ynu.ac.kr (J.L.); Tel.: +82-53-810-3812 (J.-H.L.); +82-53-810-2533 (J.L.)

Table S1. Structures and names of benzophenone derivatives. Compounds were grouped according to structural category as non-halogenated, mono-halogenated, multi-/mixed-halogenated, and fluorine-rich/CF3/perfluorinated benzophenone derivatives. MICs and biofilm formation at 50 µg/mL in 96-well plates. Purity values provided by the suppliers are included where available. A standard deviation of 0 indicates that all replicate measurements yielded identical values within the resolution of the assay; in some cases, very small variations (e.g., < 0.01) were present but were rounded to 0 due to rounding to the nearest integer.

Number	Benzophenone derivatives	Purity %	Structure	MIC (µg/mL)	Biofilm formation %
**Non-halogenated benzophenone derivatives**					
1	Benzophenone	98	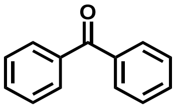	>200	100 ± 0
2	Benzophenone imine	95	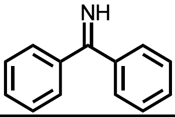	>200	98 ± 0
3	Benzophenone-2,4'-dicarboxylic acid	98	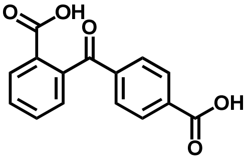	>200	99 ± 0
4	Benzophenone-4,4'-dicarboxylic acid	95	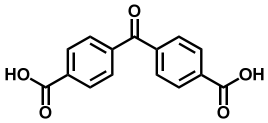	>200	98 ± 0
5	2-Aminobenzophenone	98	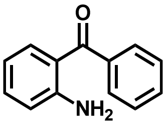	>200	31 ± 10
6	4-Aminobenzophenone	98	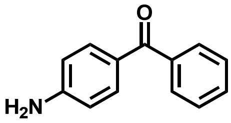	>200	72 ± 17
7	3,4-Diaminobenzophenone	95	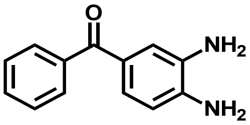	>200	27 ± 6
8	4,4'-Bis(dimethylamino)benzophenone	95	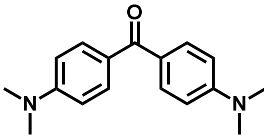	>200	99 ± 0
**9**	**2,2'-Dihydroxybenzophenone**	**98**	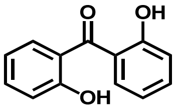	**200**	**5 ± 3**

10	4,4'-Dihydroxybenzophenone	98	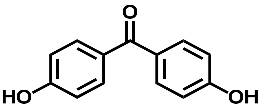	>200	99 ± 1
11	2,2',4,4'-Tetrahydroxybenzophenone	98	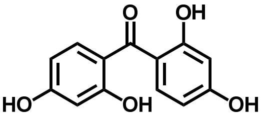	>200	100 ± 0
12	2,3,3',4,4',5'-Hexahydroxybenzophenone	98	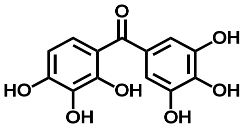	>200	99 ± 0
13	2-Hydroxy-4-N-octyloxybenzophenone	98	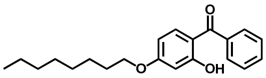	>200	99 ± 0
14	4,4'-Dimethylbenzophenone	98	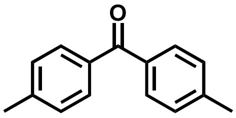	>200	100 ± 0
15	4,4'-Diphenylbenzophenone	98	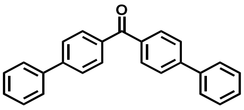	>200	58 ± 37
**Mono-halogenated benzophenone derivatives**					
16	(4-Aminophenyl)(4-fluorophenyl)methanone	97	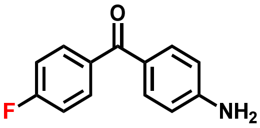	>200	60 ± 20
17	2-Amino-2'-fluoro-5-nitrobenzophenone	98	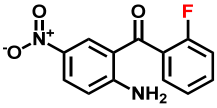	>200	99 ± 0
18	2-Amino-4'-chlorobenzophenone	98	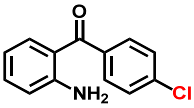	>200	46 ± 48
19	2-Amino-4'-fluorobenzophenone	97	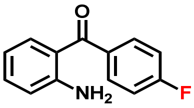	>200	100 ± 0
20	2-Amino-5-bromobenzophenone	97	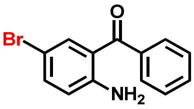	>200	10 ± 2
21	2-Amino-5-chlorobenzophenone	98	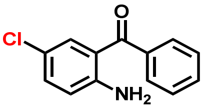	>200	53 ± 51
22	2-Amino-5-fluorobenzophenone	95	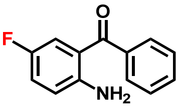	>200	61 ± 19
23	2-Amino-5-iodobenzophenone	95	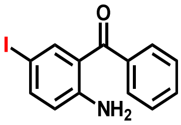	>200	94 ± 14

24	2-Bromobenzophenone	97	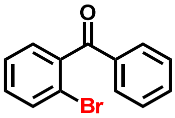	>200	12 ± 2
25	3-Bromobenzophenone	98	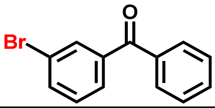	>200	99 ± 0
26	4-Bromobenzophenone	97	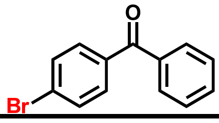	>200	99 ± 0
27	3-Bromo-4'-methoxybenzophenone	98	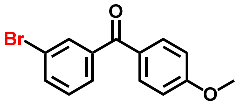	>200	92 ± 8
28	4-Bromo-2'-methylbenzophenone	97	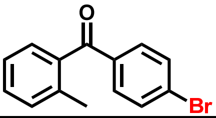	>200	99 ± 0
29	4-Bromo-4'-methoxybenzophenone	98	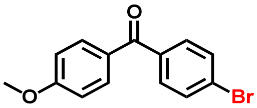	>200	99 ± 0
30	4-Bromo-4'-nitrobenzophenone	98	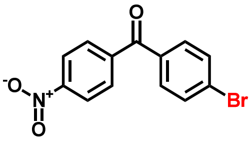	>200	34 ± 3
31	5-Bromo-2-hydroxybenzophenone	97	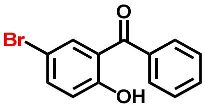	>200	99 ± 0
32	2-Chlorobenzophenone	98	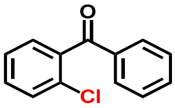	>200	13 ± 5
33	3-Chlorobenzophenone	98	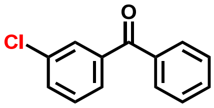	>200	100 ± 0
34	4-Chlorobenzophenone	98	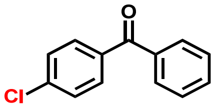	>200	100 ± 0
35	2-Chloro-5-nitrobenzophenone	98	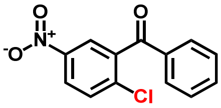	>200	10 ± 1
36	4'-Chloro-3,4-dihydroxybenzophenone	99	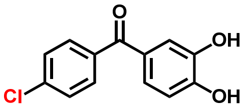	200	48 ± 18
37	4-Chloro-3-nitrobenzophenone	97	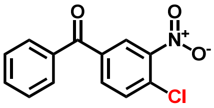	>200	60 ± 44

**38**	**4-Chloro-4'-hydroxybenzophenone**	97	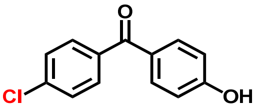	**100**	**3 ± 3**
39	4-Chloro-4'-methoxybenzophenone	98	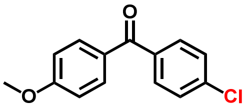	>200	100 ± 0
40	4-Chloro-4'-nitrobenzophenone	97	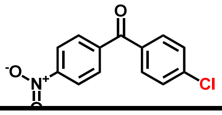	>200	84 ± 28
41	5-Chloro-2-(methylamino)benzophenone	97	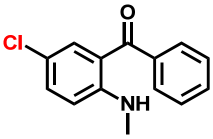	>200	98 ± 0
42	5-Chloro-2-hydroxy-4-methylbenzophenone	95	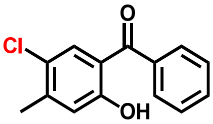	>200	98 ± 0
43	5-Chloro-2-hydroxybenzophenone	98	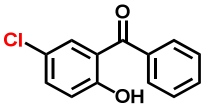	>200	55 ± 19
44	2-Fluorobenzophenone	98	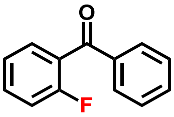	>200	59 ± 24
45	3-Fluorobenzophenone	98	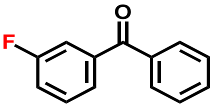	>200	100 ± 0
46	4-Fluorobenzophenone	98	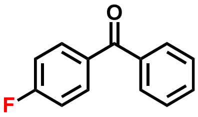	>200	100 ± 0
47	4-Fluoro-4'-hydroxybenzophenone	98	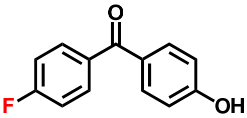	>200	39 ± 7
48	4-Fluoro-4'-methylbenzophenone	98	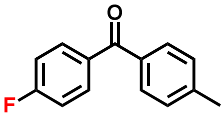	>200	99 ± 0
49	4-Fluoro-4'-nitrobenzophenone	98	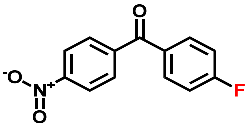	>200	61 ± 30
50	2-Iodobenzophenone	97	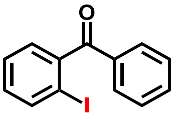	>200	60 ± 41
51	4-Iodobenzophenone	97	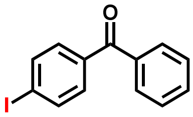	>200	99 ± 0

	**Multi-/mixed/halogenated and other halogen-containing derivatives**				
52	2-Amino-2',5-dichloro-benzophenone	98	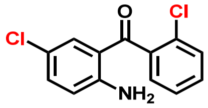	>200	21 ± 8
53	2-Amino-2'-chloro-5-ni-trobenzophenone	98	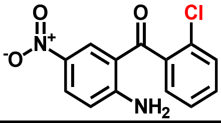	>200	53 ± 7
54	2-Amino-5-bromo-2'-chlorobenzophenone	98	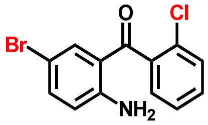	>200	33 ± 4
55	2-Amino-5-bromo-2'-fluorobenzophenone	98	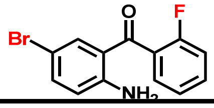	>200	11 ± 3
56	2-Amino-5-chloro-2'-fluorobenzophenone	98	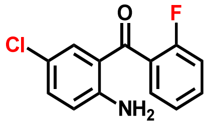	>200	19 ± 4
57	[3-(Bromomethyl)phe-nyl](phenyl)methanone	98	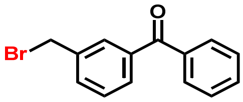	200	80 ± 29
58	3-Bromo-4'-fluorobenzo-phenone	98	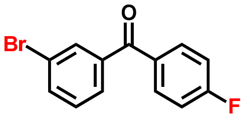	>200	99 ± 0
59	4-(Bromomethyl)benzo-phenone	98	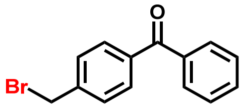	>200	99 ± 0
60	4-Bromo-3'-fluorobenzo-phenone	98	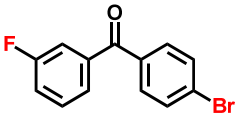	>200	99 ± 0
61	4-Bromo-4'-chloroben-zophenone	98	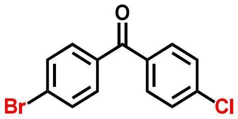	>200	92 ± 18
62	4-Bromo-4'-fluorobenzo-phenone	98	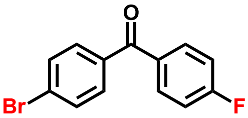	>200	98 ± 1
63	(5-Bromo-2-chloro-phenyl)(4-ethoxy-phenyl)methanone	98	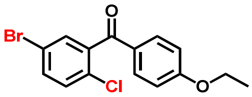	>200	98 ± 0
64	2-Chloro-4'-fluoroben-zophenone	95	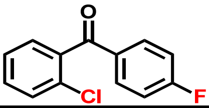	>200	27 ± 11

65	2,2'-Dichlorobenzophenone	98	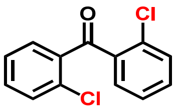	>200	58 ± 22
66	2,4-Dichlorobenzophenone	97	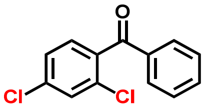	>200	79 ± 24
67	2,4'-Dichlorobenzophenone	98	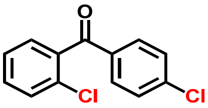	>200	65 ± 29
68	2,5-Dichlorobenzophenone	98	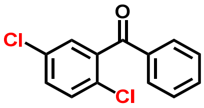	>200	68 ± 24
69	2',5-Dichloro-2-hydroxybenzophenone	98	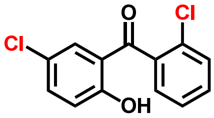	>200	56 ± 26
70	3,4-Dichlorobenzophenone	95	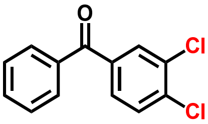	>200	100 ± 0
71	3,4,4'-Trichlorobenzophenone	95	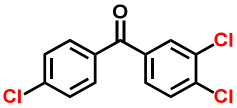	>200	100 ± 0
72	4-Chloromethylbenzophenone	97	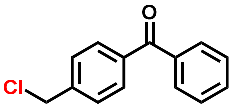	>200	22 ± 9
73	4,4'-Dichlorobenzophenone	98	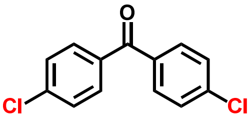	>200	100 ± 0
74	4'-Chloro-5-fluoro-2-hydroxybenzophenone	97	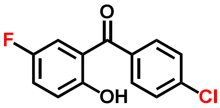	>200	59 ± 45
75	2,3-Difluorobenzophenone	95	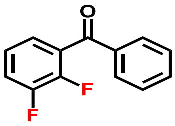	>200	53 ± 24
76	2,4-Difluorobenzophenone	98	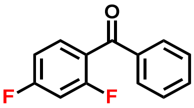	>200	98 ± 0
77	2,4'-Difluorobenzophenone	96	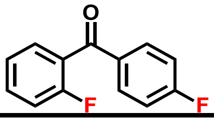	>200	98 ± 1
78	2,5-Difluorobenzophenone	95	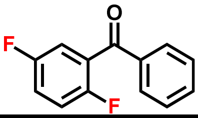	>200	64 ± 16

79	2,6-Difluorobenzophenone	95	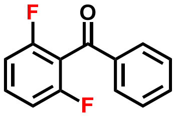	>200	25 ± 9
80	3,3'-Difluorobenzophenone	98	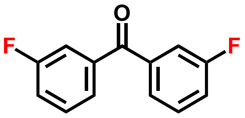	>200	76 ± 14
81	3,4-Difluorobenzophenone	97	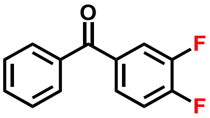	>200	100 ± 0
82	3,5-Difluorobenzophenone	95	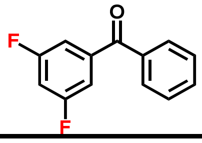	>200	100 ± 0
83	4,4'-Difluorobenzophenone	98	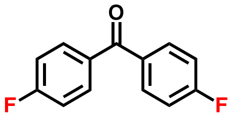	>200	99 ± 1
84	3-Nitro-4-chloro-4'-fluorobenzophenone	97	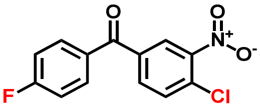	>200	99 ± 0
**Fluorine-rich, trifluoromethyl, and perfluorinated derivatives**					
**85**	**Decafluorobenzophenone**	**97**	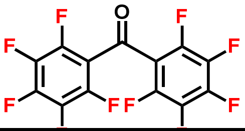	**>200**	**2 ± 0**
86	2-Fluoro-3-(trifluoromethyl)benzophenone	97	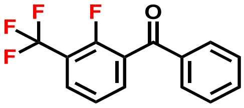	>200	100 ± 0
87	2-Fluoro-4-(trifluoromethyl)benzophenone	95	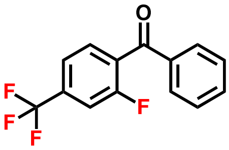	>200	100 ± 0
88	2-Fluoro-5-(trifluoromethyl)benzophenone	95	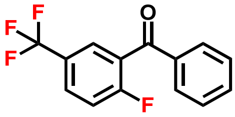	>200	100 ± 0
89	2-Fluoro-6-(trifluoromethyl)benzophenone	95	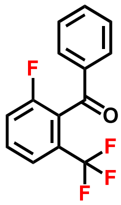	>200	54 ± 37
90	3-Fluoro-4-(trifluoromethyl)benzophenone	96	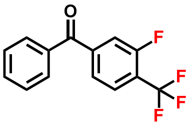	>200	80 ± 33

91	3-Fluoro-5-(trifluoromethyl)benzophenone	97	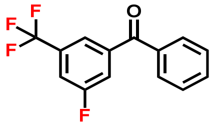	>200	100 ± 0
92	4-Fluoro-3-(trifluoromethyl)benzophenone	97	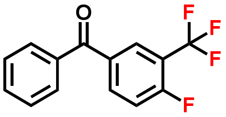	>200	99 ± 0
93	2,3,4,5,6-Pentafluorobenzophenone	98	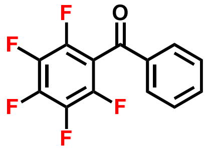	>200	94 ± 11
94	3,4,5-Trifluorobenzophenone	97	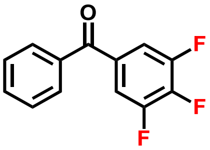	>200	97 ± 7
95	2-(Trifluoromethyl)benzophenone	98	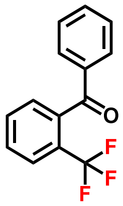	>200	99 ± 1
96	3-(Trifluoromethyl)benzophenone	95	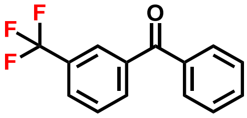	>200	100 ± 0
97	4-(Trifluoromethyl)benzophenone	95	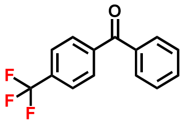	>200	100 ± 0
98	3,3'-Bis(trifluoromethyl)benzophenone	99	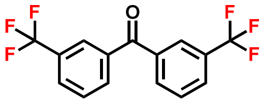	>200	99 ± 0
99	3,4'-Bis(trifluoromethyl)benzophenone	98	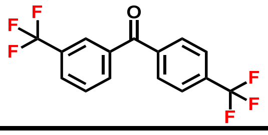	>200	99 ± 0
100	3,5-Bis(trifluoromethyl)benzophenone	97	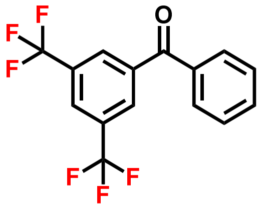	>200	99 ± 0
101	4-Methoxy-4'-trifluoromethylbenzophenone	97	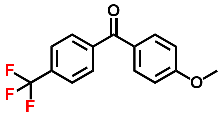	>200	99 ± 0

Table S2. ***In silico*** **ADMET prediction of selected benzophenone derivatives**.

**Parameters**	**Benzophenone (#1)**	**4-Chloro-4'-hydroxybenzophenone (#43)**	**2,2'-Dihydroxybenzophenone (#57)**	**Decafluorobenzophenone (#75)**
Human Intestinal Absorption (HIA)	100	95.99	91.66	100
Caco-2 permeability	50.48	10.16	20.78	56.80
MDCK permeability	59.49	33.49	40.76	52.93
GI absorption	High	High	High	Low
BBB Penetration	1.70	1.72	1.50	2.75
Plasma Protein Binding	95.02	100	93.22	100
LogP (Consensus)	2.93	3.1	2.32	5.85
TPSA	17.07	37.3	57.53	17.07
P-gp inhibition	No	No	No	Inhibitor
P-gp substrate	No	No	No	No
CYP1A2 inhibition	Yes	Yes	Yes	No
CYP2C19 inhibition	Yes	Yes	No	No
CYP2C9 inhibition	No	Yes	Yes	No
CYP2D6 inhibition	No	No	No	No
CYP3A4 inhibition	No	No	No	No
CYP3A4 substrate	Weakly	No	No	Substrate
Skin permeability (logKp)	-1.59	-2.32	-3.08	-3.09
Water solubility (Pure)	37.65	16.91	160.39	0.94
Water solubility (ESOL)	Soluble	Moderately soluble	Soluble	Moderately soluble
Water solubility (Ali)	Soluble	Moderately soluble	Moderately soluble	Moderately soluble
Water solubility (Silicose-IT)	Moderately soluble	Moderately soluble	Soluble	Poorly soluble
Ames Test	mutagen	mutagen	mutagen	mutagen
Mouse carcinogenicity	negative	negative	negative	negative

Rat carcinogenicity	negative	negative	negative	Positive
hERG inhibition	medium_risk	medium_risk	medium_risk	medium_risk
Acute algae toxicity	0.095	0.042	0.063	0.008
Acute fish toxicity (medaka)	0.030	0.006	0.032	0.001
Acute fish toxicity (minnow)	1.59E-02	4.90E-03	1.51E-02	4.39E-06
Rat IP LD50 Classification	Non Toxic in AD	Class 5 in AD	Class 5 in AD	Class 4 in AD
Rat IV LD50 Classification	Class 4 in AD	Class 4 in AD	Class 4 in AD	Class 4 in AD
Rat Oral LD50 Classification	Class 5 in AD	Class 5 in AD	Class 5 in AD	Class 4 in AD
Rat SC LD50 Classification	Class 4 in AD	Class 5 in AD	Class 5 in AD	Class 4 in AD
Lipinski Rule	0	0	0	1
Ghose rule	0	0	0	1
Egan rule	0	0	0	1
Muegge rule	2	0	0	2
Veber rule	0	0	0	0
Brenk alerts	0	0	0	2
PAINS alerts	0	0	0	0
Lead-likeness	1	2	1	2

Table S3. Primer sequences used for qRT-PCR.

Gene	Function	Sequences
ALS3	Cell surface adhesion protein; biofilm formation, epithelial adhesion	Forward 5'-CAA CAT CAA CCA ACC AAT CTC-3'<br>Reverse 5'-TGA ATA ACA GAA CCA GAT CCG-3'
ECE1	Candidalysin peptide toxin; epithelial damage and immune response activation	Forward 5'-CCA GAA ATT GTT GCT CGT GTT GCC A-3'<br>Reverse 5'-TCC AGG ACG CCA TCA AAA ACG TTA G-3'
EFG1	Transcriptional regulator of biofilm formation, hyphal growth, and virulence	Forward 5'-CAT AAA CCT CAT CAC AAG TGG ATT G-3'<br>Reverse 5'-AAA GAT TGG AAG AAC CCA TCA ACT-3'
ERG9	Squalene synthase; ergosterol biosynthesis and sterol precursor production	Forward 5'-AAA ATG GGT AAT GGT ATG GC-3'<br>Reverse 5'-ACT TGG GGA ATG GCA CAA AA-3'
ERG11	Sterol 14α-demethylase; ergosterol biosynthesis and fungal membrane function	Forward 5'-AAG AAT CCC TGA AAC CAA-3'<br>Reverse 5'-CAG CAG CAG TAT CCC ATC-3'
HWP1	Hyphal cell wall adhesin; biofilm formation, host colonization, and virulence	Forward 5'-TGG TGC TAT TAC TAT TCC GG-3'<br>Reverse 5'-CAA TAA TAG CAG CAC CGA AG-3'
RBT5	Heme-binding surface protein; heme-iron utilization, biofilm formation, and virulence	Forward 5'-CTG CTG AAA GTT CTG CAC CA-3'<br>Reverse 5'-GCT TCA ACG GAA ACA GAA GC-3'
UCF1	Filamentous growth-associated protein; cAMP signaling, biofilm induction, and fluconazole response	Forward 5'-ATG GCG GGA AAG AAA AAG TC-3'<br>Reverse 5'-CCC AAG TTT CAT CAC GAA CA-3'
UME6	Zn(II)2Cys6 transcription factor; filamentous growth regulation and environmental response	Forward 5'-AGC ACC AAA TTC GCC TTA TG-3'<br>Reverse 5'-AGG TTG AGC TTG CTG CAG TT-3'

YWP1	Secreted yeast cell wall protein; immune evasion, adhesion, and biofilm formation	Forward 5'-GTT CCA TTT TTC CAA GTT CAT TTA G-3'<br>Reverse 5'-TCA AGA GTA GAA CCT TCA AGA GCA G-3'
ZAP1	Zinc-responsive transcription factor; zinc homeostasis and transcriptional regulation	Forward 5'-CGA CTA CAA ACC ACC AGC TTC ATC-3'<br>Reverse 5'-CCC CTG TTG CTC ATG TTT TGT T-3'
RDN18	Housekeeping	Forward 5'-AGA AAC GGC TAC CAC ATC CCA-3'<br>Reverse 5'-CGA ATG GGC CCT GTA TCG T -3'

Table S4. Molecular docking binding energies of benzophenone derivatives against *C. albicans* Als3 (PDB: 4LEB). Five independent runs using PharmaCoNet. Digitalin, epigallocatechin, diazepam, and rutin were used as positive controls.

Molecular Docking Binding Energy (kcal/mol)						
**Name**	**Run 1**	**Run 2**	**Run 3**	**Run 4**	**Run 5**	**Mean ± SD**
Digitalin	-6.38	-5.76	-6.06	-6.17	-6.04	-6.08 ± 0.22
4-Chloro-4′-hydroxybenzophenone	-4.99	-4.92	-4.99	-4.89	-5.06	-4.97 ± 0.07
Epigallocatechin	-4.90	-4.97	-4.81	-5.02	-4.88	-4.92 ± 0.08
2,2′-Dihydroxybenzophenone	-4.47	-4.42	-4.41	-4.42	-4.45	-4.43 ± 0.03
Diazepam	-5.57	-5.55	-5.58	-5.59	-5.58	-5.57 ± 0.02
Benzophenone	-4.31	-4.31	-4.30	-4.29	-4.30	-4.30 ± 0.01
Rutin	-5.90	-5.83	-5.52	-5.97	-5.87	-5.82 ± 0.17
Decafluorobenzophenone	-3.02	-3.02	-3.03	-3.01	-3.02	-3.02 ± 0.01

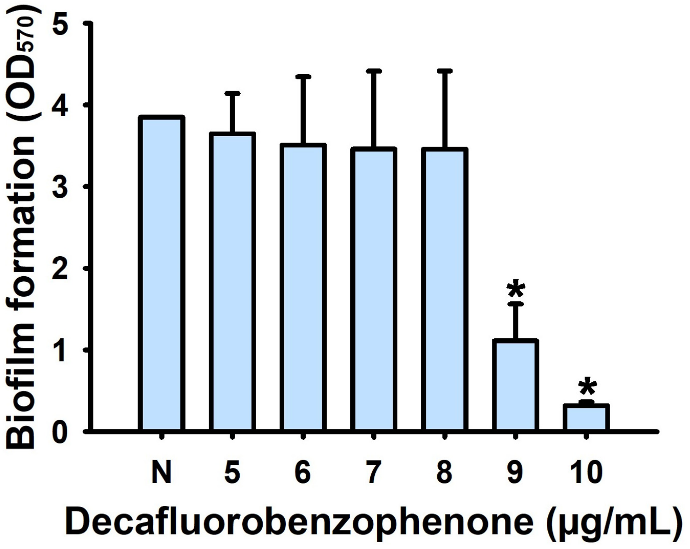

Figure. S1. Determination of the IC_{50} value of decafluorobenzophenone against *C. albicans* DAY185 biofilm formation. Biofilm inhibition was evaluated at concentrations of 5-10 μg/mL. Sigmoidal dose–response curve fitting was performed using SigmaPlot (Systat Software, San Jose, CA, USA), and the estimated IC_{50} value was approximately 8.7 μg/mL.

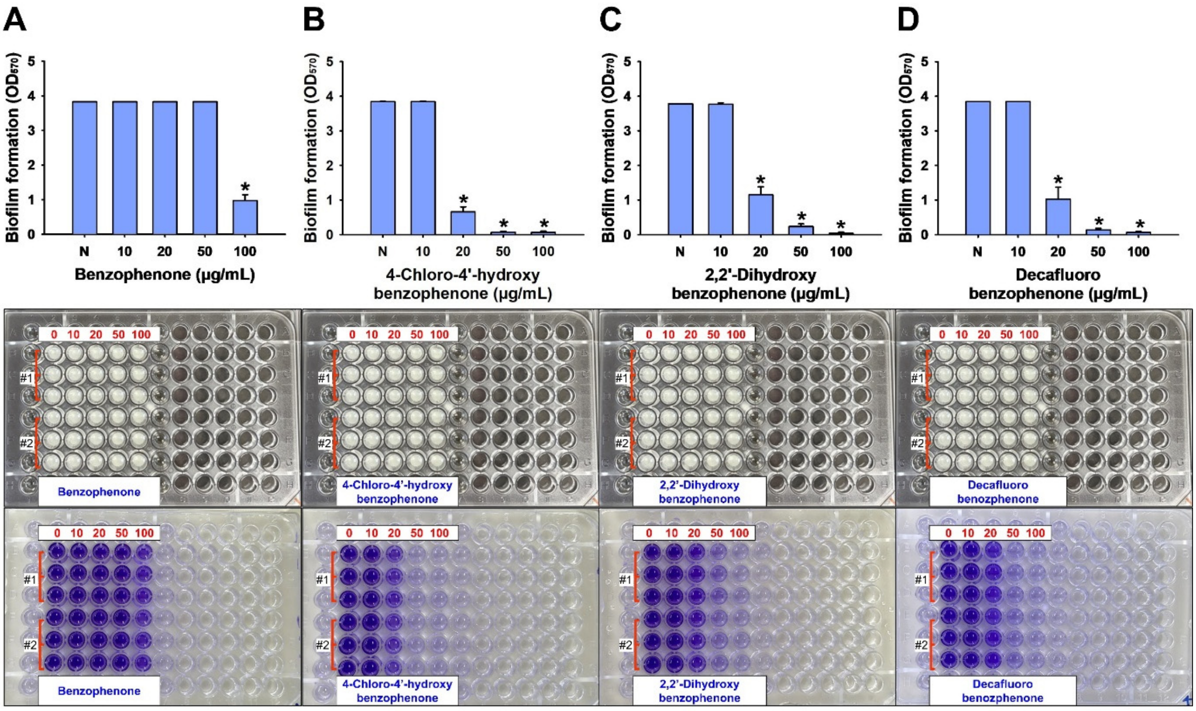

Figure. S2. Antibiofilm activity of halogenated benzophenone derivatives against azole-sensitive *C. albicans* ATCC 10231. Backbone benzophenone(A), 4-chloro-4′-hydroxybenzophenone (B), 2,2′-dihydroxybenzophenone (C), and decafluorobenzophenone (D). Compounds were tested at concentrations of 0-100 μg/mL. *, $p < 0.05$ compared to untreated controls (N).

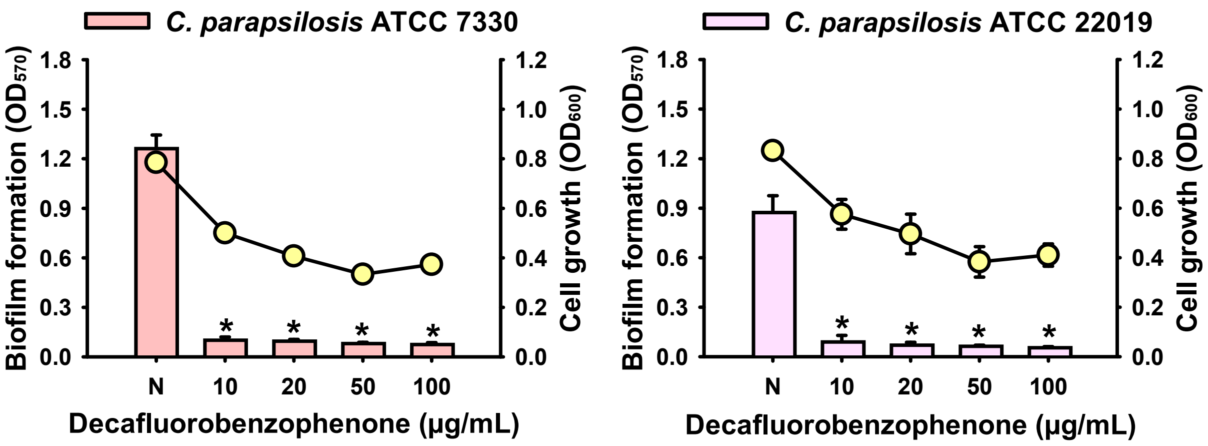

Figure. S3. Antibiofilm activity of decafluorobenzophenone against *Candida parapsilosis* strains. Biofilm formation of *C. parapsilosis* ATCC 7330 and ATCC 22019 was evaluated after treatment with decafluorobenzophenone at concentrations of 10-100 µg/mL. Decafluorobenzophenone significantly reduced biofilm formation in both *C. parapsilosis* strains in a concentration-dependent manner. Data are presented as mean ± standard deviation. *$P < 0.05$ compared with untreated controls (N).

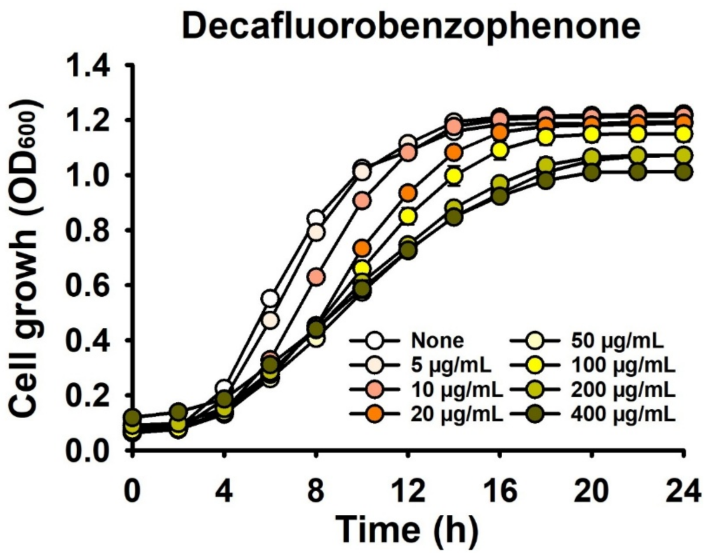

Figure. S4. Growth curves of *C. albicans* DAY185 at different concentrations (5, 10, 20, 50, 100, 200, and 400) of decafluorobenzophenone. OD_{600} was measured every 2 h over 24 h.

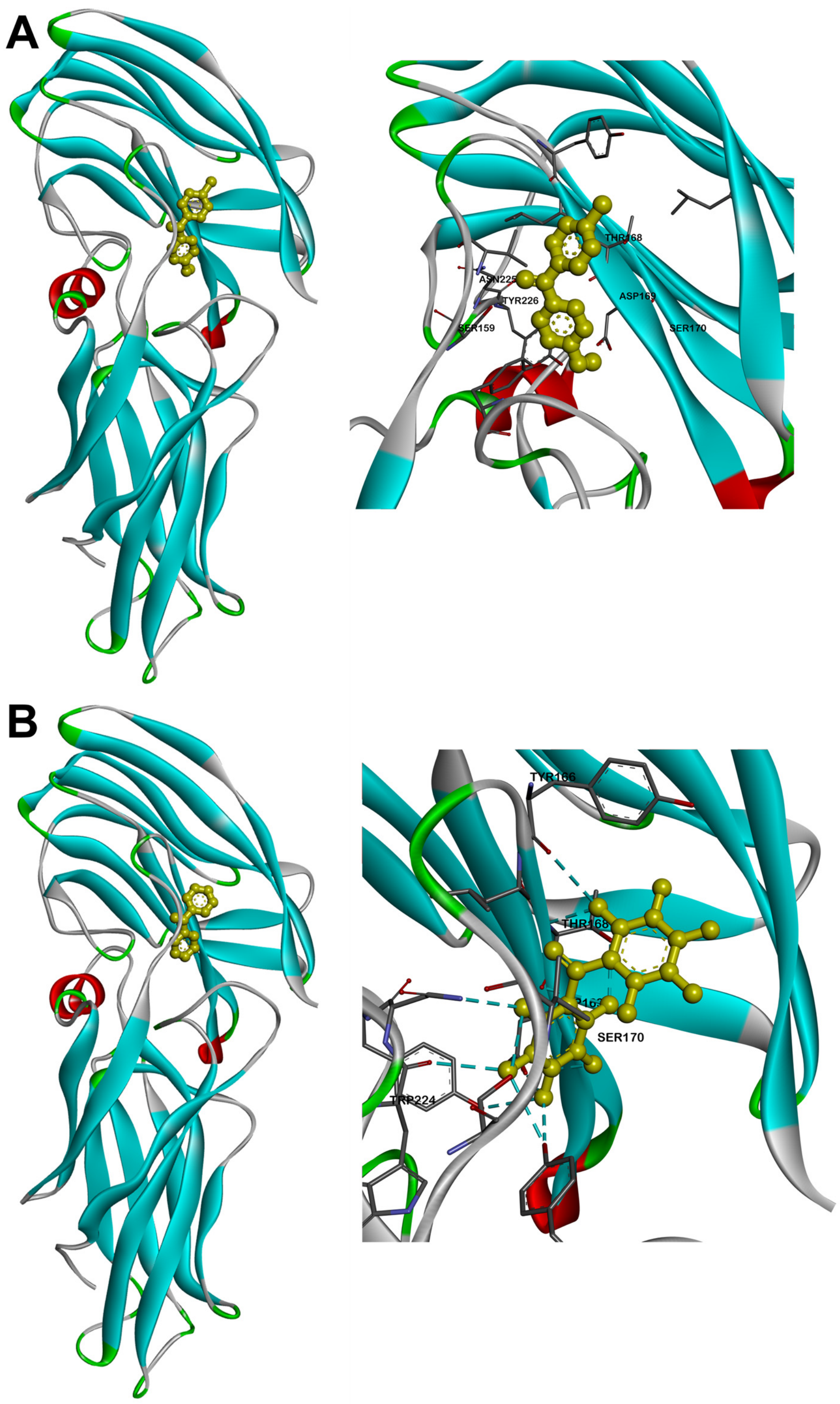

Figure. S5. Three-dimensional molecular docking analysis in the binding pocket of Als3 for 4-chloro-4′-hydroxybenzophenone (A) and decafluorobenzophenone (B).

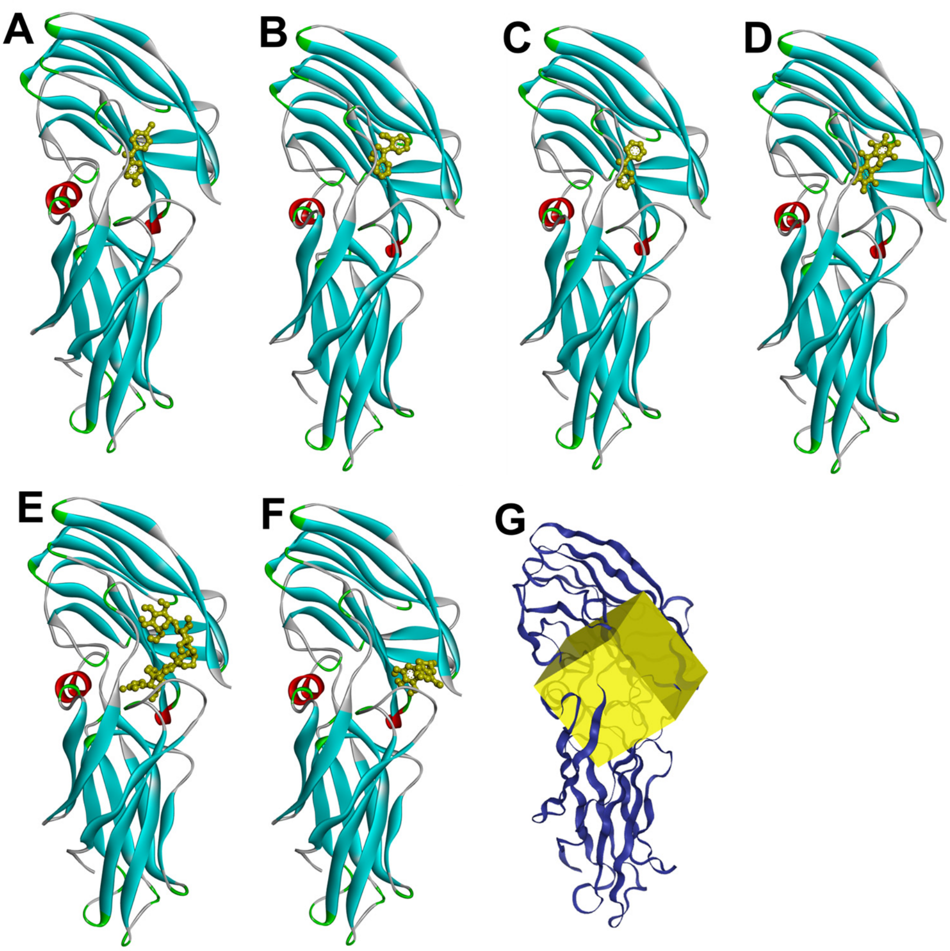

Figure. S6. Three-dimensional Als3 protein–ligand interactions of 4-chloro-4′-hydroxybenzophenone (A), 2,2′-dihydroxybenzophenone (B), benzophenone (C), decafluorobenzophenone (D), digitalin (E), epigallocatechin (F), and predicted binding pocket by Pharmaco-Net platform. Digitalin and epigallocatechin were used as positive controls+.
